# Predictive models of the genetic bases underlying budding yeast fitness in multiple environments

**DOI:** 10.1093/nargab/lqag084

**Published:** 2026-08-11

**Authors:** Kenia E Segura Abá, Paulo Izquierdo, Gustavo de Los Campos, Melissa D Lehti-Shiu, Shin-Han Shiu

**Affiliations:** Genetics and Genome Sciences Graduate Program, Michigan State University, East Lansing, MI 48824,United States; Department of Energy Great Lakes Bioenergy Research Center, Michigan State University, East Lansing,MI 48824,United States; Department of Plant and Environmental Sciences, New Mexico State University, Las Cruces, NM 88003,United States; Genetics and Genome Sciences Graduate Program, Michigan State University, East Lansing, MI 48824,United States; Department of Epidemiology and Biostatistics, Michigan State University, East Lansing, MI 48824,United States; Department of Statistics and Probability, Michigan State University, East Lansing, MI 48824,United States; Institute for Quantitative Health Science and Engineering, Michigan State University, East Lansing, MI 48824,United States; Department of Plant Biology, Michigan State University, East Lansing, MI 48824,United States; Genetics and Genome Sciences Graduate Program, Michigan State University, East Lansing, MI 48824,United States; Department of Energy Great Lakes Bioenergy Research Center, Michigan State University, East Lansing,MI 48824,United States; Department of Plant Biology, Michigan State University, East Lansing, MI 48824,United States; Department of Computational Mathematics, Science, and Engineering, Michigan State University, East Lansing, MI 48824,United States

## Abstract

The ability of organisms to adapt and survive depends on the effects of genes and the environment on fitness. However, the multigenic nature of fitness and genotype-by-environment interactions hinder our understanding of the genetic basis of fitness. Here, we established fitness prediction models for 35 environments using machine learning and existing fitness data and different genetic variant types for a *Saccharomyces cerevisiae* population. Models revealed that the predictive ability of genetic variants varied across environments, with copy number variants explaining the majority of fitness variation in most cases. Model interpretation showed that different variant types identified distinct gene sets associated with predictive variants. These gene sets were significantly enriched in experimentally validated genes affecting fitness in only a subset of environments, indicating that many genes influencing fitness remain unexplored. Notably, non-experimentally validated genes were more important than validated ones for fitness predictions. Gene contributions to predictions were both isolate- and environment-dependent, pointing to gene-by-gene and gene-by-environment interactions. Furthermore, models uncovered experimentally validated and novel candidate genetic interactions for a well-characterized stress, the fungicide benomyl. These findings highlight the feasibility of identifying the genetic basis of fitness by using different genetic variant types and offer novel targets for future functional analysis.

## Introduction

Deciphering the connection between phenotypic and genetic variation is a long-standing challenge in biology [1]. The influence of genetic factors on phenotypes varies between traits, environments, and populations. Some traits are controlled by a single gene [2] while others are multigenic [3], and most traits are influenced by both genetic and environmental factors [4, 5]. Furthermore, genetic interactions (e.g. epistasis) and genotype-by-environment interactions also contribute substantially to trait variation [6–15]. This complexity frequently results in a non-linear relationship between genotype and phenotype. Thus, prediction of complex traits from genomic data and identification of causal genes remain challenging tasks [16–19].

Quantitative trait locus (QTL) analyses and genome-wide association studies (GWAS) are widely used to uncover genetic variants underlying phenotypic variation [20–23]. More recently developed genomic prediction methods [24, 25], building upon the principles of QTL analyses, are able to predict complex traits and have led to significant advancements in the fields of crop and animal breeding and human genetics [16, 26, 27]. Examples of successful applications of genomic prediction include accelerating breeding programs for dairy cattle [28, 29] and wheat [30] and predicting coronary heart disease risk in humans [31]. In contrast to QTL analyses and GWAS, genomic prediction methods based on machine learning are able to capture non-linear relationships between genetic variants and multi-omics data [19, 32]. For example, transcriptomic data, single nucleotide polymorphism (SNP) genotypes, and methylation data have been used to predict flowering time and to identify interactions between data types [33]. Transcriptomic data and SNP genotypes have also been combined with environmental data to predict grain yield in wheat [34] and maize [35].

In addition, machine learning-based genomic prediction models can be further interpreted to provide hypotheses of the genetic bases of complex traits [36–39]. Such interpretation falls into two main categories: global and local [36]. Global interpretation strategies measure the overall contribution of individual predictor variables (features, e.g. a SNP) to trait prediction, whereas local interpretation strategies quantify feature contribution to predicted trait values for each individual in a population. Model interpretation strategies have been used to, e.g. understand long non-coding RNA functions in humans [40], identify plant flowering time genes [33], and investigate vertebrate enhancer activity [41].

Interpretable machine learning-based genomic prediction models allow a better understanding of the genotype-to-phenotype association, which has been facilitated by recent population genome sequencing projects. These projects have generated genotype and phenotype data from a relatively larger number of genetically distinct individuals compared to earlier studies in, e.g. human [42–46], *Drosophila melanogaster* [47], *Arabidopsis thaliana* [48–50], and *Saccharomyces cerevisiae* [51–54]. In *S. cerevisiae* (budding yeast), a pangenome of 1011 natural and laboratory isolates is available [51]. Furthermore, these isolates have been phenotyped for fitness, a complex trait relevant to adaptation and biotechnology applications, under 35 different environmental conditions [51]. They have also been genotyped at SNPs, and structural variants—e.g. copy number variants (CNVs) and presence/absence variants (PAVs)—have been identified. These types of genetic variants have been shown to be associated with phenotypic variation in various species, including humans, livestock, plants, and yeast [55–62]. Together with the rich functional annotation available for budding yeast, the fitness and variant datasets provide a valuable resource for further assessing the impact of environment and types of genetic variants on trait predictions.

Here, we aim to understand the genetic basis of fitness in a natural population of *S. cerevisiae* grown in 35 environments by establishing genomic prediction models using a published dataset [51]. Using a subset of 750 diploid isolates, we assessed how well fitness is predicted using SNPs, PAVs, and CNVs in different environments. By interpreting the genomic prediction models, we identified SNP, PAV, and CNV features contributing the most to model performance and quantified their contributions both locally (at the level of individual isolates) and globally in each environment. In addition, we assessed how well our models identified benchmark genes validated in published studies. Finally, we asked what genetic interactions can be discovered by different genetic variant types and how much these interactions contribute to predictions in specific environments.

## Materials and methods

### Data pre-processing, kinship calculation, and estimation of population structure

Three types of genetic variant data—SNPs, open reading frames (ORFs) of PAVs, and ORF CNVs—and fitness measurements (File S1) for 750 diploid *S. cerevisiae* isolates grown in 35 different environments were obtained from [51]. Here, fitness is the ratio between the size of colonies grown in an environment and that in the reference environment (YPD medium at 30°C). The SNP data were filtered using VCFtools v0.1.16 [63] to retain only bi-allelic SNPs with a minor allele frequency (MAF) > 5% and missing data < 20%, using the parameters “–maf 0.05,” “–max-alleles 2,” “–min-alleles 2,” “–max-missing 0.2,” “–recode,” and “–remove-indels.” The final genotype dataset used in this study included 118 382 SNPs (File S2). Genotypes were re-coded into fastPHASE format with PLINK v1.9 [64, 65] using the parameter “–recode12 fastphase.” Missing genotypes were imputed using fastPHASE v1.4.8 with the parameter “-T10” [66]. Genotypes were encoded as {−1, 0, 1} corresponding to {AA, Aa, aa}, where A is the major allele and a is the minor allele. Kinship between isolates was calculated using the centered identity-by-state method [67] implemented in TASSEL v5 [68] with the parameters “-KinshipPlugin” and “-method Centered_IBS” (File S3). Population structure was modeled as the first five principal components (PCs) of the SNP genotypes estimated with the Scikit-learn v1.2.2 [69] Principal Component Analysis function (File S4). Eighty-eight ORFs with missing PAV and CNV values in all isolates were excluded, resulting in PAV and CNV datasets with 7708 ORFs out of the original 7796 reported by [51] (Files S5 and S6, respectively).

### Predictive modeling of fitness in each environment using genomic prediction

For each of the 35 environments, a “single-environment” model was built where the complete set of SNP, PAV, CNV, or PC values (referred to as features) was used to predict fitness with linear and non-linear methods. Three linear models were implemented in R v4.3.2: Ridge Regression Best Linear Unbiased Predictor (rrBLUP) using rrBLUP v4.6.3 [70], Bayesian-Least Absolute Shrinkage and Selection Operator (Bayesian LASSO) [71], and BayesC [72]. Bayesian LASSO and BayesC were implemented using BGLR v1.1.4 [73] with 32 000 iterations, where the first 3200 iterations were discarded as burn-in. Non-linear, machine learning regression models were implemented in Python 3.11.5 using Scikit-learn v1.2.2 for Random Forest (RF) [74] and XGBoost v2.0.3 [75] for eXtreme Gradient Boosting (XGBoost) [75].

Before building any model, one-sixth of the yeast isolates were randomly held out as the test set, which was used exclusively to evaluate model performance. The remaining five-sixths of the isolates, referred to as the training set, were used to train the models. For rrBLUP models, the rrBLUP R package automatically estimates the regularization and kernel parameters from the data, so no hyperparameter tuning was conducted with cross-validation. No hyperparameter tuning was conducted for BayesC or BayesianLASSO either. rrBLUP, BayesC, and Bayesian LASSO training were conducted within a five-fold cross-validation scheme and repeated 20 times. For machine learning algorithms, hyperparameter tuning was conducted within a five-fold cross-validation scheme, which was repeated 10 times for RF and 100 times for XGB, to account for the larger hyperparameter space in XGB. The hyperparameters for RF, which were tuned using Scikit-Learn’s GridSearchCV function, were “max_depth” [3, 5, 10], “max_features” [0.1, 0.5, “sqrt,” “log_2_,” “None”], and “n_estimators” [100, 500, 1000]. The hyperparameters for XGBoost, which were tuned using HyperOpt v0.2.7 [76], were “learning_rate” [0.01–0.4], “max_depth” [2–10] with a step size of 1, “subsample” [0.5–1.0], “colsample_bytree” [0.7–1], and “n_estimators” [5–500] with a step size of 5. To choose the best parameter combinations, we used the negative mean squared error for RF in GridSearchCV and the negative mean *R*^2^ for XGBoost in HyperOpt. The best combination of parameters was chosen based on the validation set performance and used to train a new model within a five-fold cross-validation scheme. The coefficient of determination (*R*^2^) was calculated between the observed and predicted relative fitness values, and the average of the 20 training repetitions was used as the validation set performance of RF and XGB.

### Feature importance and feature selection

The impact of features on the performance of the RF models was assessed using two methods. The first was Gini importance [77]. For SNPs, the top {2*^n^* | *n* ∈ *ℤ*, 1 ≤* n *≤ 10} ⋃ {1000 × *n* | *n* ∈ ℤ, 1 ≤ *n* ≤ 30} features, where *n* is the number of features, were selected based on average Gini importance values, which we refer to as “Gini importance.” The Gini importance was calculated by taking the average feature importance across the 20 training repetitions of the RF models built using the complete feature sets. These feature subsets were used to build new RF models to find the minimum feature set size required to reach peak training performance (referred to as an optimized RF model). For PAV and CNV features, the top {2*^n^* | *n* ∈ ℤ, 1 ≤ *n* ≤ 10} ⋃ {250 × *n* | *n* ∈ ℤ, 1 ≤ *n* ≤ 30} features were selected. The optimized single-environment RF models were selected based on the inflection point of a feature selection curve (*x*-axis: number of features, *y*-axis: average performance *R*^2^ across 10 training iterations on the validation set). These feature subsets were then used to build models for all other algorithms. Gini importance values for SNP, PAV, and CNV features for RF models using the complete feature sets can be found in File S7.

SHAP v.0.42.1 [39, 78] was also used to assess feature importance. SHapley Additive exPlanations (SHAP) values for each feature were estimated for each isolate used for training RF models on the complete or optimized feature sets. Because training was repeated 20 times for RF models built using complete feature sets and 10 times for optimized RF models, the model with the highest validation performance, which was assumed to represent the data the best, was used to estimate SHAP values. The SHAP values for SNPs, PAVs, and CNVs from the RF models trained on the complete feature sets are provided in File S7.

### Predicting model performances using technical and fitness-related features

The effects of the features narrow-sense heritability (*h^2^*) of fitness, median fitness (med), and fitness variance (var) on performance ($y$) of the 35 PC-, SNP-, PAV-, or CNV-optimized single-environment RF models were estimated by ordinary least squares (OLS) regression using the statsmodels v0.14.2 Python package. Feature effects were estimated using the following equation:


\begin{eqnarray*}
y &=& {\beta _0} + {\beta _1}{x_h} + {\beta _2}{x_m} + {\beta _3}{x_v} + {\beta _4}{x_h}{x_m}\\&+& {\beta _5}{x_h}{x_v}\,\, + {\beta _6}{x_m}{x_v} + {\beta _7}{x_h}{x_m}{x_v} + e,
\end{eqnarray*}

where $\beta $ is a vector of coefficients and $\beta $_0_ is the intercept; *x* is a vector of values of the features *h^2^* (*h*), med (*m*), and var (*v*); and $e$ is a vector of residuals. An additional OLS regression model was built using the number of features used to train the optimized RF models. Regression models were built to assess the performance of the optimized RF models built with one genetic variant type (i.e. PCs, SNPs, PAVs, or CNVs). Linear model goodness of fit was evaluated using both the R^2^ and adjusted R^2^, where the latter corrects the R^2^ for the number of predictor variables relative to the sample size to prevent overestimating model fit [79]. SHAP values were estimated for each linear model using the LinearExplainer function to further assess feature effects on model performances.

The *h*^2^ of fitness in all 35 environments was estimated using a mixed model equation implemented in the Sommer v4.3.0 R package [80] using the mmer function. The formula for the mixed model was as follows:


\begin{eqnarray*}
{y_i} = {Z_i}{u_i} + {e_i},
\end{eqnarray*}

where *y_i_* is a vector of fitness values, *u_i_* is a vector of random effects, *e*_i_ are residuals for environment *i* (*i* = 1, …, 35), and *Z_i_* is an incidence matrix of random effects. A 750 × 1 vector of trait values for environment *i* was used to represent *y_i_*. A 750 × 750 additive relationship matrix and a 750 × 750 dominance relationship matrix were estimated using the SNP genotypes and used as random effects.

### Mapping of SNPs and ORFs to genes

The *S. cerevisiae* reference genome (S288C; version R64-3-1) and gene annotation files (gff3) were obtained from the *Saccharomyces* Genome Database (SGD; http://sgd-archive.yeastgenome.org/sequence/S288C_reference/) and used to obtain the list of S288C genes and their translational start and stop positions. SNP variants were assigned to genes if they were found in genic regions (File S8). ORFs comprising PAV and CNV features were assigned to S288C genes based on reciprocal best match in two steps. In the first step, sequence similarity between query ORF nucleotide sequences and the protein sequences of S288C was determined using BLASTx [81, 82] with parameters “-max_target_seqs 2,” “-max_hsps 1,” and “-evalue 1e-06.” The top matching S288C gene, G, was considered a candidate gene to a query ORF, O, if the percent identity was ≥95% and the *E*-value was <1e-6.

In the second step, ORF-to-gene mapping was finalized by using the nucleotide sequence of the candidate S288C gene, G, identified in the first step as the query to search against non-S288C ORF nucleotide sequences using tBLASTx with the same parameters as the first step. If the top match of an S288C gene G remained the ORF O, based on the same filter criteria in the first step, O was mapped to G. Out of 7708 ORFs, 5902 mapped to 5873 unique S288C genes, and 16 of these ORFs mapping to >1 gene were excluded. To obtain systematic gene names for the mapped genes, identifiers of genes from the non-redundant database were mapped to the S288C gene identifiers using the NCBI Datasets Gene tool (https://www.ncbi.nlm.nih.gov/datasets/gene/) and the SGD YeastMine Gene List tool (https://yeastmine.yeastgenome.org/yeastmine/bag.do). The ORF-to-gene mapping data can be found in File S9.

### Feature rank percentile correlations

Spearman’s rank correlation between the Gini importance and the average absolute SHAP values of SNP, PAV, or CNV features from the optimized RF models was determined after dropping features with zero importance values. The remaining features were ranked using the Pandas “DataFrame.rank” function and the “average” method, and the correlation was calculated based on the features common to both importance measures. Similarly, for correlations based on the RF models trained on the complete feature sets, features with zero importance values were excluded, and the remaining features were ranked.

To assess relationships between SNP and PAV or CNV features from the optimized RF models, these features were mapped to genes and were ranked according to either the maximum Gini importance or the maximum average absolute SHAP value (calculated across the isolates) of all the features that mapped to the same genic region (see “Mapping of SNPs and ORFs to genes” section). SNP, PAV, and CNV features that did not map to any gene, including intergenic SNPs and/or had a zero Gini importance or average absolute SHAP value, were excluded from the ranking. The Spearman’s rank correlation was calculated between SNPs versus PAVs or CNVs, and PAVs versus CNVs.

To assess relationships between environments from the optimized RF models, we determined the number of overlapping genes across the 35 environments. Features from RF models trained on the optimized SNP, PAV, or CNV feature sets were mapped to genes. To determine the number of environments in which a gene appeared within the optimized feature set, genes with non-zero feature importance—the highest average absolute SHAP value or the highest Gini importance of the features mapped to that gene—were assigned a value of 1, creating a gene presence matrix. These presence values were summed across all environments, and the resulting counts were compared to a null distribution of median counts generated from 10 000 permutations of the gene presence matrix in which each column (environment) in the gene presence matrix was permuted individually. The actual distribution of environment counts was compared to the randomized null distribution using the Kolmogorov–Smirnov test (alternative = “greater”).

Spearman’s rank correlation of shared features between two environments was estimated after dropping features with non-zero importance in at least one environment and ranking the remaining features for each environment. Correlations were determined for ranks based on Gini importance or average absolute SHAP values of shared features.

### Benchmark fitness genes and known genetic interactions

Candidate known fitness genes were obtained from SGD by searching for “benomyl,” “caffeine,” “copper(II) sulfate” (CuSO_4_), and “sodium arsenite” (also known as sodium meta-arsenite). Phenotype annotations of candidate genes found in the “Chemicals” category page for these compounds were filtered by the “Phenotype” and “Mutant Information” values based on the following criteria. A candidate gene was considered as a benchmark fitness gene for the target condition if it had mutant information annotations matching “null Allele,” “reduction of function,” or “reduction of function Allele” and phenotype annotations matching “resistance to chemicals: decreased,” “viability: decreased,” “metal resistance: decreased,” “oxidative stress resistance: decreased,” “respiratory growth: decreased,” or “stress resistance: decreased.” After the filtering step, 386 genes with experimental evidence of an effect on fitness compared to a reference condition were identified for benomyl, 752 for caffeine, 162 for CuSO_4_, and 280 for sodium meta-arsenite. S288C genes with mapped SNP features included 370/386 benomyl fitness genes, 725/752 caffeine fitness genes, 156/162 CuSO_4_ fitness genes, and 278/280 sodium meta-arsenite fitness genes. The ORF features mapped to 350/386 benomyl fitness genes, 688/752 caffeine fitness genes, 145/162 CuSO_4_ fitness genes, and 270/280 sodium meta-arsenite fitness genes. In addition, a list of manually curated genes for benomyl (5 genes), caffeine (10 genes), CuSO_4_ (15 genes), and sodium meta-arsenite (15 genes) were obtained from the literature.

Experimentally validated genetic interaction information for S288C was collected from the BioGRID database (https://thebiogrid.org/). Gene pairs with the evidence annotations “Synthetic Growth Defect,” “Synthetic Lethality,” “Synthetic Rescue,” “Negative Genetic,” and “Positive Genetic” were selected, totaling 438 546 genetic interactions. Additional genetic interactions that were experimentally verified using single and double mutant yeast strains grown under a control condition (glucose) or in 30 µg/ml benomyl were obtained from data published by Costanzo *et al*. [83]. These genetic interactions observed under control conditions or in the presence of benomyl were filtered according to a stringent confidence threshold (*P* < .05 and |ε| > 0.12, where ε is the genetic interaction score), yielding 3417 genetic interactions in the control condition and 3472 genetic interactions in the presence of benomyl. The combined set of unique genetic interactions (including BioGRID and [83] data) resulted in 441 520 gene pairs (File S10).

### Gene Ontology term, metabolic pathway, benchmark gene, and experimentally validated genetic interaction enrichment analyses

Gene Ontology (GO) term annotations v.20220912 were retrieved from the GO GAF v2.2 format (sgd.gaf; http://current.geneontology.org/annotations/index.html). GO terms with experimental evidence codes (IDA, IPI, IMP, IGI, IEP, HDA, HMP, HGI, HEP) were kept and mapped to the features. Pathway annotations (downloaded 10 October 2022) were retrieved from the MetaCyc database (https://metacyc.org/group?id=biocyc14-55140-3843260367). Before conducting enrichment analyses for GO terms and pathways, SNP, PAV, and CNV features were mapped to the S288C genes as detailed in “Mapping of SNPs and ORFs to genes” section. Gene features that met the feature selection cut-off criteria were referred to as “important genes” for predicting fitness in an environment, i.e. these features were used to train the optimized RF models. For features that did not meet the feature selection cut-off, the genes they mapped to were considered as the background (unimportant) gene set. Features that did not map to any genes or mapped to multiple genes were excluded from the analysis.

Enrichment of important genes for a GO term or a pathway annotation, *A*, was determined by calculating four values in a 2 × 2 contingency table: *a—*the number of important genes with *A, b—*the number of background genes with *A, c—*the number of important genes without *A*, and *d—*the number of background genes without *A*. For each annotation, these four values were used to determine the enrichment *P* with two-sided Fisher’s exact tests. To correct for multiple testing, hypothesis tests with *P *= 1 were removed, and the remaining *P-*values were corrected using the Benjamini and Hochberg method [84].

For analysis of benchmark fitness gene enrichment in each environment, we tested whether benchmark genes related to that environment (i.e. benomyl benchmark genes were identified within the YPD Benomyl 500 μg/ml model) or to a different environment (e.g. benomyl benchmark genes were identified within the YPD Caffeine 40 mM model) were enriched at different rank percentile thresholds (within the top 1%, 5%, 10%, 15%, 20%, or 25% of ranked genes) of Gini importance or average absolute SHAP values for each optimized model. Benchmark genes associated with benomyl, caffeine, copper(II) sulfate, and sodium meta-arsenite stress were sourced from both SGD and the literature (see “Benchmark fitness genes and known genetic interactions” section). Enrichment was assessed among genes within the top 1%, 5%, 10%, 15%, 20%, or 25% rank percentile based on SNP, PAV, or CNV features. Features were mapped to genes, and rank percentiles were calculated using either the highest Gini importance or the highest average absolute SHAP value of the features mapped to that gene. Features that had non-zero importance, did not map to a gene, or mapped to multiple genes were excluded from the rankings. To ensure that all genes in the yeast genome represented by the SNP, PAV, or CNV features were included in the analysis, gene rankings from the optimized single-environment RF models were combined with rankings of non-overlapping genes from models trained on the complete feature sets. Enrichment analysis was conducted for five environments (YPD Caffeine 40 mM, YPD Caffeine 50 mM, YPD Benomyl 500 μg/ml, YPD CuSO_4_ 10 mM, and YPD Sodium meta-arsenite 2.5 mM) in a similar manner to the GO term and pathway enrichment analyses. However, the annotation *A* represents whether the gene is a benchmark fitness gene in either the benomyl, caffeine, copper(II) sulfate, sodium meta-arsenite, or literature-based gene lists.

### Assessing the contribution of benchmark genes to fitness predictions

To assess the importance of benchmark genes for fitness predictions, we trained 45 new RF models for predicting fitness using reduced SNP, PAV, or CNV feature sets for five environments: YPD Caffeine 40 mM, YPD Caffeine 50 mM, YPD Benomyl 500 μg/ml, YPD CuSO_4_ 10 mM, and YPD Sodium meta-arsenite 2.5 mM. Three kinds of reduced feature sets were generated: (i) a feature set containing important non-benchmark genes (identified by the optimized single-environment RF models) plus benchmark genes (from the RF models trained on the original complete feature sets), (ii) only important non-benchmark genes, and (iii) only benchmark genes. SNP, PAV, or CNV features from the RF models built using complete feature sets and the optimized RF models were mapped to genes (see “Mapping of SNPs and ORFs to genes” section). Features were obtained by representing each gene by the SNP within the genic region, PAV, or CNV feature with the highest average absolute SHAP importance. Then, benchmark and non-benchmark genes were determined based on their presence in the SGD benchmark gene lists. Intergenic SNPs were excluded. RF models were trained using Scikit-learn v1.2.2 and evaluated as described under “Predictive modeling of fitness in each environment using genomic prediction” section. The number of features and validation and testing performances for these models are provided in File S11.

### Analyzing genetic distance to S288C and the effect on benchmark gene importance

Genetic distances (Euclidean distances) between the 625 diploid isolates from the training set and S288C were estimated from SNP and PAV values. SNP genotypes were re-coded to {0, 1, 2} genotype encodings, where 0 refers to a genotype homozygous for the reference allele, 1 for heterozygous, and 2 for homozygous for the alternative allele (File S12). S288C SNP genotypes were denoted as a vector of 0s, since S288C is the reference genome and was not one of the training isolates. S288C PAV values were determined from the BLASTx and reciprocal tBLASTx results outlined in “Mapping of SNPs and ORFs to genes” section, where an ORF was given a 0 value if it did not map to the S288C genome, or a 1 if it did. Euclidean distance between pairs of isolates was estimated using the scipy.spatial.distance.pdist function. No scaling was applied to the SNP or PAV matrices prior to calculating Euclidean distances. The distance matrices are provided in Files S13 and S14 for SNPs and PAVs, respectively.

To compare the maximum absolute SHAP values of benchmark genes between two clusters of isolates, *K*-means clustering was performed on the SNP and PAV genetic distance matrices using the KMeans function from Scikit-learn v1.2.2. For each distance matrix, the number of clusters (*k*) was varied from 2 to 10, and elbow plots were used to select the optimal number of clusters (*k* = 6 for SNPs, *k* = 4 for PAVs) based on inertia. No scaling was applied before clustering. The cluster containing S288C was identified, and the remaining clusters represent isolates that are the least genetically related to S288C. A two-sided Mann–Whitney U test (alternative = “greater”) was used to compare the distributions of median absolute SHAP values of benchmark genes between these two clusters, using the Mann–Whitney U test as implemented in SciPy v1.11.4. To construct these distributions, SHAP values were obtained from either the optimized RF model or the complete RF model trained on all SNP or PAV features for a given environment. Only SHAP values corresponding to features that mapped to benchmark genes were retained. For each isolate in a cluster, the median absolute SHAP value was computed per gene.

### Clustering of SHAP values

Based on the median absolute SHAP values of the top 20 SNP, PAV, or CNV features from the optimized RF models, budding yeast isolates were clustered for five environments: YPD Caffeine 40 mM, YPD Caffeine 50 mM, YPD Benomyl 500 μg/ml, YPD CuSO_4_ 10 mM, and YPD Sodium meta-arsenite 2.5 mM. Hierarchical clustering was performed using the scipy.cluster.hierarchy.linkage function with the “ward” method and “euclidean” distance metric. To obtain clusters with different granularities, clusters were split by distance thresholds tailored to SNPs, PAVs, and CNVs for the YPD Caffeine 40 mM, YPD Caffeine 50 mM, YPD Benomyl 500 μg/ml, YPD CuSO_4_ 10 mM, and YPD Sodium meta-arsenite 2.5 mM environments (see File S15). Features were also clustered for visualization purposes. SHAP values of the top 20 SNP, PAV, and CNV features for each model are provided in File S16.

To assess the relationship between the SHAP values of isolates and fitness across clusters, the median absolute SHAP value across the top 20 features was calculated for each isolate in a cluster (*x* variable). Similarly, the median fitness of isolates was calculated for each cluster (*y* variable). A linear regression line was fitted between *x* and *y* using the scipy.stats.linregress function with default arguments. The function provided estimates of the slope, intercept, standard error of the intercept, Pearson correlation, and the *P*-value for a hypothesis test of whether the slope is zero (based on the Wald test using the *t*-distribution).

### Estimating SHAP interaction values

To estimate SHAP interaction values between feature pairs, three new RF models and three new rrBLUP models were built to predict fitness in YPD Benomyl 500 μg/ml using reduced SNP, PAV, or CNV feature sets. An additional four RF models were trained using integrated feature sets (SNP + PAV, SNP + CNV, PAV + CNV, or SNP + PAV + CNV). The reduced feature sets consisted of features that mapped to benomyl benchmark genes. The feature with the highest average absolute SHAP value was selected to represent each gene. Intergenic SNPs were excluded. These reduced SNP, PAV, and CNV feature sets were then concatenated column-wise to create the integrated feature sets. Of the 118 382 available SNP features, 370 were selected. Of the 7708 PAV and CNV features, 350 were selected for each. RF models (implemented in Scikit-learn v1.5.2) and rrBLUP models were trained as described in “Predictive modeling of fitness in each environment using genomic prediction” section. SHAP interaction values were estimated from the RF model with the highest validation *R*^2^ among training repetitions for each reduced or integrated feature set. SHAP interaction values between gene features were calculated using the shap.TreeExplainer.shap_interaction_values function (implemented in SHAP v.0.42.1).

## Results and discussion

### Genetic variants differ in their ability to explain variation in fitness across environments

The availability of genome-wide SNPs, ORF PAVs, and ORF CNVs and growth traits measured in 35 different environments for *S. cerevisiae* isolates published by Peter *et al*. [51] provided an opportunity to compare levels of genetic variation across genetic variant types, which may differ in their ability to explain fitness trait variation. We analyzed 750 diploid isolates with genomic and phenotypic information in all 35 environments. To gauge the levels of genetic variation, we estimated SNP MAF, the percent presence of ORFs across isolates, and CNV distributions across isolates. All three genetic variant types exhibited substantial variation. MAF values ranged from 0.01 to 0.80 (mean = 0.20 ± 0.13, Supplementary Table S1), ORFs were present in an average of 78% ± 40% of isolates (Supplementary Table S1), and CNV values of ORFs were biased toward lower copy numbers (mean CNV value per ORF = 0.88 ± 2.33, Supplementary Table S1). Together, these results indicate extensive genetic diversity across isolates.

**Figure 1.**
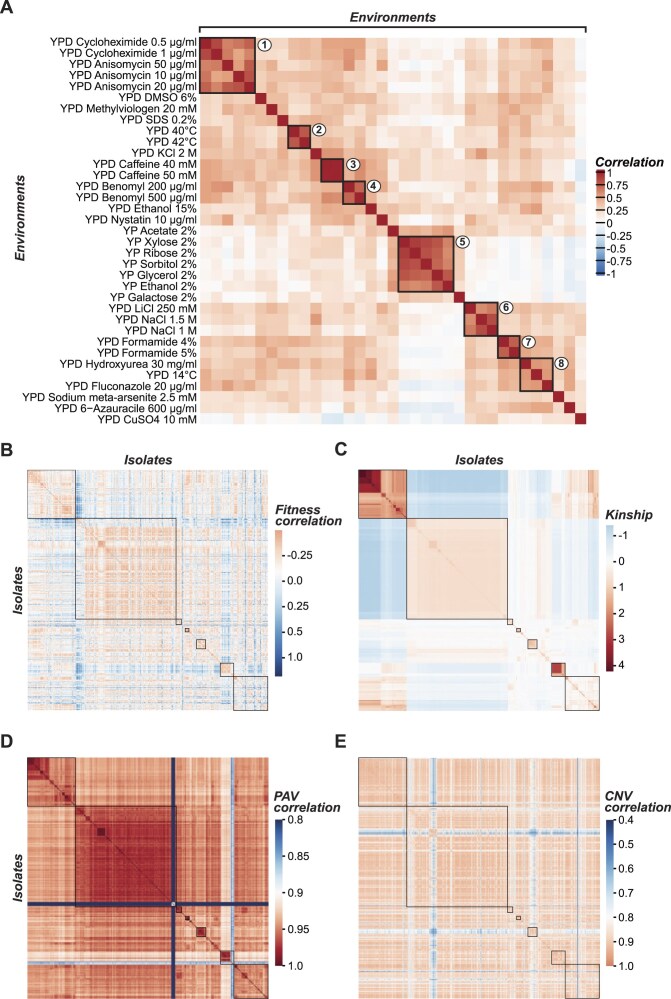
Phenotypic correlation between environments based on fitness values and relationships between yeast isolates based on fitness and genotype data. (**A**) Relationships between fitness values for pairs of environments shown as Pearson’s correlation coefficients (correlation). Colors indicate degrees of correlation. Clusters (rectangles labeled 1–8) were determined via hierarchical clustering based on the Euclidean distances of yeast isolate fitness values across environments. Relationships between 750 diploid yeast isolates based on (**B**) correlations of fitness across 35 environments, (**C**) kinship (calculated using SNPs), (**D**) correlations of PAV profiles, and (**E**) correlations of CNV profiles. Kinship was used to order isolates and identify clusters of isolates (black boxes).

Furthermore, the effects of genetic variants on fitness often depend on the environment, leading to variability in fitness for a single genetic background across environments [51, 85]. To better understand how fitness responses correlate across environments, we used normalized colony sizes (colony size relative to that in a control environment) as an approximation of fitness [51]. An environment is defined as a treatment with a specific temperature or a chemical at a specific concentration (Supplementary Table S1). Pearson’s correlation coefficients (*r*) were estimated between fitness values of pairs of environments and clustered using Euclidean distance (mean *r* = 0.24 ± 0.19, Fig. 1A). We found eight environment clusters (*r* > 0.51) encompassing 24 of 35 environments (Fig. 1A). Cluster 1 was indicative of similar cellular response mechanisms—anisomycin and cycloheximide inhibit peptide chain elongation by binding to different subunits of the ribosome [86, 87]. Environments within the remaining clusters except cluster 8 tended to be similar temperatures (cluster 2: 40°C and 42°C), to be the same chemicals but at different concentrations (clusters 3, 4, and 7: caffeine, benomyl, and formamide, respectively), or to have shared chemical properties (cluster 5: xylose, ribose, sorbitol, glycerol, and ethanol; cluster 6: LiCl and NaCl). Although the environments in cluster 8 do not have obvious relationships, there are likely similar genetic mechanisms underlying differences in fitness among isolates in these environments.

While the presence of environment clusters is expected because isolates respond similarly to related treatments, there are substantial differences in fitness values between related isolates across environments (*r* = 0.59 ± 0.20, Fig. 1B), raising the question of the extent to which genetic variation across budding yeast isolates explains fitness differences across environments. To address this, we estimated genetic relatedness using three types of genetic variants—SNPs (i.e. kinship, Fig. 1C), PAVs (Fig. 1D), and CNVs (Fig. 1E)—and fitness correlations of isolates across environments. The kinship matrix reveals clusters indicative of population structure (rectangles, Fig. 1C) that are partially preserved in a matrix of fitness correlations (Fig. 1B), and we found that the variation in fitness across environments is correlated with population structure (*r* = 0.23, *P* < 2.2 × 10^–16^, Supplementary Fig. S1A). This pattern also holds for PAV profile correlations (*r* = 0.20, *P* < 2.2 × 10^–16^, Supplementary Fig. S1B), but it is not as clear for CNV profiles (Fig. 1E), which were lowly correlated with kinship (*r* = 0.09, *P* < 2.2 × 10^–16^, Supplementary Fig. S1C). Thus, fitness is expected to be more highly correlated with PAV correlations (*r* = 0.15, *P* < 2.2 × 10^–16^, Supplementary Fig. S1D) than with CNV correlations (*r* = 0.13, *P* < 2.2 × 10^–16^, Supplementary Fig. S1E). Furthermore, PAV correlations and CNV correlations were lowly correlated (*r* = 0.06, *P* < 2.2 × 10^–16^, Supplementary Fig. S1F). These findings indicate that the genetic component contributing to variation in fitness across environments is composed of different genetic variant types contributing overlapping and distinct information that can be used to predict fitness in different environments.

### Genetic variant type influences model performance in fitness prediction

To determine how well different types of genomic variants predict yeast fitness in a given environment, we built single-environment fitness prediction regression models using five algorithms encompassing three linear (BayesC, Bayesian Least Absolute Shrinkage Operator, and rrBLUP) and two non-linear (RF and XGBoost) approaches. SNPs, PAVs, or CNVs were used as input features (see “Materials and methods” section). For each environment and algorithm, “baseline” models were constructed using the first five PCs of the SNP data as a proxy of population structure (capturing 59% of the genetic variation), and “optimized” models were generated using a feature selection approach to maximize prediction accuracy (see “Materials and methods” section). Model performance was assessed by calculating the coefficient of determination between true and predicted fitness values (*R*^2^, Supplementary Table S2). RF testing set performances for the optimized models tended to be better than those of the other algorithms for CNVs (Supplementary Table S3). Additionally, RF models are easily interpretable with local interpretation methods; therefore, we base subsequent analyses in this paper on the RF models.

**Figure 2.**
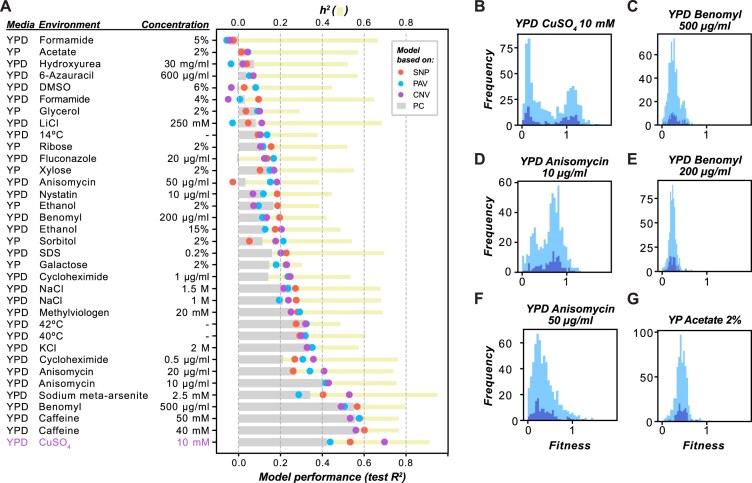
Narrow-sense heritability, model performance, and fitness distributions across environments. (**A**) Narrow-sense heritability (*h*^2^, mean = 0.59 ± 0.17) estimates and the test set performances (*R*^2^) of the optimized RF models for each environment. Single-environment models were built with PCs (approximate population structure; gray bars), SNPs (orange), PAVs (blue), or CNVs (purple). The YPD CuSO_4_ 10 mM model using CNV features achieved the highest test performance. Distribution of fitness values for the (**B**) YPD CuSO_4_ 10 mM, (**C**) YPD Benomyl 500 μg/ml, (**D**) YPD Anisomycin 10 μg/ml, (**E**) YPD Benomyl 200 μg/ml, (**F**) YPD Anisomycin 50 μg/ml, and (**G**) YPD Acetate 2% environments. Light and dark blue histograms represent fitness value distributions of the training and test sets, respectively.

Different types of genetic variants have distinct, environment-dependent contributions to fitness prediction. Regardless of algorithm, the *R*^2^ of models based on population structure alone varied greatly between environments, ranging from 0 to 0.56 (PCs, median *R*^2 ^= 0.15 and mean *R*^2 ^= 0.20, Fig. 2A, Supplementary Table S2). In 32 environments, SNP, PAV, and/or CNV information improved models compared with those based solely on population structure (Fig. 2A). Surprisingly, despite the correlation between CNV and fitness variation being lower than that between SNP or PAV and fitness (Supplementary Fig. S1A–C), the CNV-based model performance (median *R*^2 ^= 0.21) was similar to that using SNPs (median *R*^2 ^= 0.20) or PAVs (median *R*^2 ^= 0.18). This seemingly contradictory result is likely due to the higher degree of dependence of SNPs and PAVs on population structure relative to that of CNVs, suggesting that CNVs may identify candidate fitness genes that differ from those identified by population structure. Utilizing CNVs to identify candidate fitness genes is feasible because they have been reported to have large deleterious effects on fitness that may also vary depending on the genetic background and the environmental condition [51, 59, 62, 88, 89]. Furthermore, the higher performance of CNV-based models compared to the SNP- and PAV-based models in certain environments is consistent with the finding by Peter *et al*. [51] that CNVs explain more trait variation than SNPs on average.

Using models established with the RF algorithm, fitness values in three environments (YPD Caffeine 40 mM, YPD Caffeine 50 mM, and YPD Benomyl 500 μg/ml) were predicted well by three feature types (SNPs, PAVs, and PCs) (Fig. 2A), and the highest prediction performance was observed for YPD CuSO_4_ 10 mM with CNV features (*R*^2 ^= 0.70, Fig. 2A). SNP-, PAV-, CNV-, and PC-based models performed the best in 34.2% (12 out of 35), 22.9% (8 out of 35), 37.1% (13 out of 35), and 5.7% (2 out of 35) of the environments, respectively. CNV-based models outperformed SNP-, PAV-, and PC-based models in 51.4% (18 out of 35), 51.4%, and 62.9% (22 out of 35) of environments, respectively. In particular, CNV-based models outperformed the PC-based models by 1.66-fold for YPD CuSO_4_ 10 mM and 1.56-fold for YPD Sodium meta-arsenite 2.5 mM. The higher performance of CNV-based models in certain environments may reflect the fact that CNVs lead to variation in the dosage of genes important for growth under stress conditions and may provide a selective advantage [62]. For example, haploid budding yeast strains with >1 copy of *CUP1* (copperthionein) have higher fitness than those that have only one copy [90]. On the other hand, SNP- and PAV-based models outperform CNV-based models in environments such as YPD NaCl 1.5 M and YPD Methylviologen 20 mM (Fig. 2A), suggesting that SNPs and PAVs likely contribute more to fitness in these environments than CNVs do. Whether this reflects underlying genetic mechanisms remains unclear. Lastly, a large proportion of the trait heritability in most environments remains unexplained by any individual variant type (Fig. 2A).

To assess why fitness is better predicted in some environments than in others, we established a linear model predicting the performances of the optimized single-environment RF models with three features—narrow-sense heritability (*h*^2^) of fitness, fitness variance, median fitness—along with all pairwise and three-way interaction terms (see “Materials and methods” section). The narrow-sense heritability of a trait measures the proportion of phenotypic variance that is explained by variance in additive genetic effects. Highly heritable traits may exhibit greater prediction accuracies, although prediction can still be challenging [91]. Measures of central tendency (median) and the spread (variance) are used to describe continuous distributions and trait distributions and have been shown to reflect the genetic basis of traits in some cases [92, 93]. For these reasons, we evaluated the impact of *h*^2^, fitness variance, and median fitness on model performances. These three features explained 42% (i.e. adjusted *R*^2^ = 0.42), 45%, 33%, and 59% of the variation in performance for PC, SNP, PAV, and CNV-based models, respectively (Supplementary Table S4). Different combinations of these features contributed to PC, SNP, PAV, and CNV model performances to varying degrees. PAVs and CNVs were better at predicting fitness traits with higher variance, lower median fitness, and lower fitness variance-by-*h*^2^ terms; PCs were predictive of environments with lower fitness variance-by-*h*^2^ terms; and no terms were statistically significantly correlated with SNP model performances (for statistics and term ranges, see Supplementary Table S4). To better understand which feature(s) are important for model performance, we determined SHAP values based on the same linear models, where positive values indicate that a feature increased model performance and negative values indicate that a feature decreased performance relative to the expected performance of a model trained on one environment. Based on SHAP values, *h*^2^ was the most correlated with model performances out of the fitness-related features tested (PC: *r* = 0.62, SNP: *r* = 0.64, PAV: *r* = 0.54), but it was negatively correlated with CNVs (*r* = −0.67, Supplementary Fig. S2). Since *h*^2^ was estimated from SNPs, this result is consistent with our finding that SNP profiles are correlated with PAVs but not with CNVs (Supplementary Fig. S1D and E). Because PCs are also derived from SNPs, and population structure confounds the relationship between PAVs and SNPs (Fig. 1C and D; Supplementary Fig. S1D), the correlation between *h*^2^ and PC-, SNP-, and PAV-based model performances may be driven by shared genetic information.

A factor that may influence model performance is the genetic architecture of the trait, which can be partially assessed based on the shape of the fitness distribution (e.g. number of peaks or skewness). For example, traits with bimodal distributions tend to have Mendelian inheritance [92], which indicates a simpler genetic basis. Because few variants influence fitness, these traits can be easier to predict. Environmental responses with a simple genetic basis may have high genetic similarity which would cause variants to contribute to fitness consistently across the population. Levels of genetic similarity that are similar to the fitness similarity lead to high trait heritability. Consistent with this, models of environments with the highest performances, such as YPD CuSO_4_ 10 mM, YPD Benomyl 500 μg/ml, and to a lesser extent, YPD Anisomycin 10 μg/ml, exhibited bimodal distributions of fitness (Fig. 2B–D) and had high *h*^2^ values (Fig. 2A and Supplementary Table S4). In contrast, environments with non-bimodal distributions of fitness, such as YPD Benomyl 200 μg/ml (Fig. 2E), YPD Anisomycin 50 μg/ml (Fig. 2F), and YP Acetate 2% (Fig. 2G), were not predicted well and had low *h*^2^ values (Fig. 2A and Supplementary Table S4). However, YPD Caffeine 40 and 50 mM and YPD Sodium meta-arsenite 2.5 mM were predicted well despite having non-bimodal distributions (Supplementary Fig. S3) and had high *h*^2^ values (Fig. 2A and Supplementary Table S4). In addition to fitness distributions, the number of features used to train models also influences model performance. Feature number explained 33% (adjusted *R*^2^, coefficient = 7.2 × 10^–5^, *P* = 1.7 × 10^–4^, Supplementary Table S4), 3%, and 0% of the variation in SNP-, PAV-, and CNV-based model performances, respectively. Taken together, these results indicate that model performance arises from a combination of technical factors, such as feature number, and genetic mechanisms and interactions that influence trait distribution and trait variance, with their contributions differing across genetic variant types.

### Different genetic variant types and environments uncover distinct sets of genes predictive of fitness

**Figure 3.**
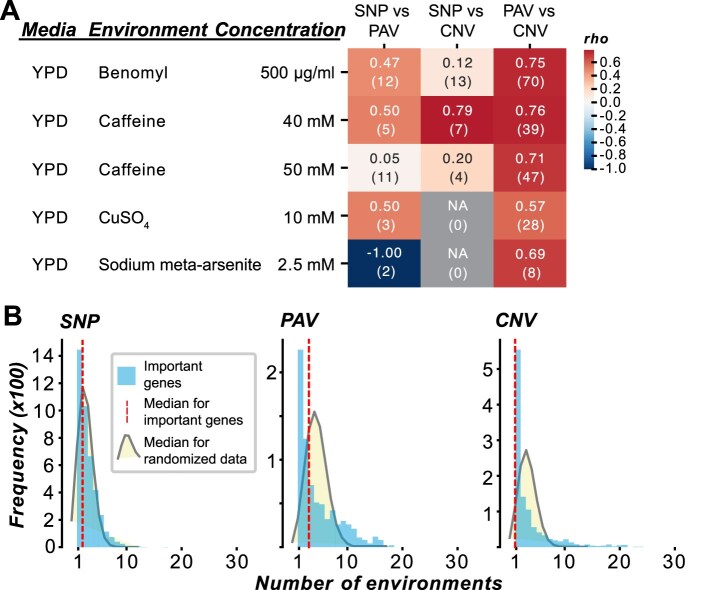
Comparisons of feature importance between variant types and across environments. (**A**) Spearman’s rank correlation (rho) between the average absolute SHAP values from the optimized RF models trained on different genetic variant types (e.g. SNP versus PAV optimized RF models’ SHAP values were compared) for the five best predicted environments. The number of genes that were shared by both feature sets is denoted in parentheses. Boxes labeled as "NA" indicate there was no overlap in important genes between feature sets. (**B**) Average absolute SHAP values from the optimized RF models were used to determine the distribution of unique or shared genes with non-zero importance across 1–35 environments (bars; dotted line: median). This distribution was compared to a null distribution of median randomized counts (see “Materials and methods” section) using the Kolmogorov–Smirnov test (alternative = “greater”); for SNPs: median *P* = 6.1 × 10^–28^; for PAVs: *P* = 1.5 × 10^–31^; for CNVs: *P* = 2.1 × 10^–71^.

To further assess the genetic basis of fitness in an environment, we determined which SNP, PAV, or CNV (i.e. features) variants contribute to fitness predictions in the optimized models for different environments using two feature importance measures—Gini importance [77] and SHAP [39, 78]. Gini importance provides a global, overall measure of feature importance among all yeast isolates, whereas SHAP values allow further exploration of feature contributions to fitness in each yeast isolate. Gini- and average absolute SHAP value-based feature rankings were significantly correlated with each other in each of the five optimized RF models with the highest performances for any genetic variant type (Supplementary Fig. S4A, Spearman’s correlation coefficients for RF models trained on complete feature sets: Supplementary Fig. S4B, Supplementary Table S5). Using the five environments where the optimized models have the best performance (Fig. 2) as examples, SHAP value-based rankings of shared features between PAVs and CNVs (i.e. ORFs that were important for predictions in both models for an environment) were significantly correlated for four environments (*P* ≤ .001, Fig. 3A, Spearman correlations based on Gini importance: Supplementary Fig. S4C, Spearman correlations for RF models trained on complete feature sets: Supplementary Fig. S4D and E, Supplementary Table S6). This overlap is expected since PAVs and CNVs are structural variants that are partially dependent on each other; however, there remains a substantial number of non-overlapping, predictive ORF features. There was little overlap in the feature importance rankings of the best five environments when comparing SNP versus PAV models and SNP versus CNV models, regardless of the feature importance measure examined (for optimized models: 3.6 × 10^–2^ ≤ *P* ≤ .9, for complete models: 1.3 × 10^–15^ ≤ *P* ≤ 1.0, Fig. 3A, Supplementary Fig. S4C–E, Supplementary Table S6).

The lack of overlap between SNPs and CNVs may stem from their different associations with genes. CNVs can span small or large segments of DNA and even entire chromosomes [88]. They can alter not only protein-coding but also regulatory regions, leading to changes in gene expression levels, which in turn can influence the regulation of other genes and downstream phenotypes, including fitness [62]. On the other hand, SNPs are point mutations that may simply be linked to the causal variants and may have a smaller impact compared to CNVs. Considering the differences in the sets of genes uncovered by these three types of features, future studies on trait variation may benefit from considering multiple types of genetic variants, in addition to SNPs, to obtain a more complete picture of the mechanisms underlying trait variation.

Our finding that related environments cluster together using fitness data (Fig. 1A) prompted us to ask if similar genetic mechanisms underlie fitness variation in related environments. To test this, we examined the overlap of predictive features across models using SHAP-based feature rankings. Among the five optimized RF models with the highest performances, YPD Caffeine 40 and 50 mM, which clustered together based on fitness values (Fig. 1A), shared 578 SNPs, 64 PAVs, and 55 CNVs (Supplementary Table S7). The rankings of these features between the two environments were significantly correlated at different strengths depending on the genetic variant (SNP: Spearman’s ρ = 0.44, *P* = 4.91 × 10^–29^; PAV: ρ = 0.85, *P* = 3.00 × 10^–19^; CNV: ρ = 0.74, *P* = 1.15 × 10^–10^), indicating that similar genetic mechanisms underlie fitness in these two environments. However, when examining the overall overlap of predictive genes across all 35 environments, we found that significantly fewer predictive genes were shared across environments than expected by random chance (Fig. 3B and Supplementary Fig. S4F), pointing to the impact of genotype-by-environment interactions and the largely unique genetic bases for fitness.

Having identified important, predictive genes in each environment, we next asked what functions these genes have that may be relevant to the environments in which they are important for fitness predictions. To explore this, we conducted GO and pathway enrichment analyses of the important genes from the optimized RF models (Supplementary Table S8, see “Materials and methods” section). We found no significantly enriched GO terms in the five optimized, best-performing RF models, but found a total of 10 enriched GO terms in eight of the remaining environments, for models using CNVs or PAVs. At the pathway level, we identified a total of 16 enriched pathways among 11 environments (Supplementary Table S8). For example, in the YPD Sodium meta-arsenite 2.5 mM model, CNV features were enriched for genes related to arsenate detoxification (odds ratio = 166.7, *q* = 0.01) and also pyridoxal 5′-phosphate biosynthesis II pathways (odds ratio = 249.7, *q* = 0.01), which are necessary for arsenic resistance [94]. SNP features for the YPD Caffeine 40 mM model were enriched for glutamine degradation I genes (odds ratio = 6.5, *q *= 0.005) and glutaminyl-tRNA^gln^ biosynthesis via transamidation genes (odds ratio = 6.5, *q *= 0.005). No functional connections between caffeine and glutamine degradation or glutamyl-tRNA biosynthesis were found in the literature. The limited enrichment of GO terms and pathway annotations likely reflects the complex nature of growth-related traits. These traits depend on the genomic and environmental contexts in which they are measured and are often highly polygenic, complicating the experimental validation of gene functions and completeness of functional annotation sets. Moreover, the experimental data underlying most GO and pathway annotations are predominantly obtained in laboratory strain genetic backgrounds. Because our study examines natural isolates, many predictive genetic variants or genes may lack functional annotations for fitness traits in the relevant environments. Nevertheless, the fact that CNVs identified genes related to arsenic resistance for the relevant YPD Sodium meta-arsenite 2.5 mM environment highlights the importance of considering structural variants to better understand the genetic basis of fitness in response to arsenic stress, and likely other complex traits.

To further explore the functions of the genes predicted as important for fitness in specific environments, we assessed whether genes experimentally verified to be important for survival in an environment (benchmark fitness genes) were enriched among the important genes in the five best predicted environments—YPD Caffeine 40 mM, YPD Caffeine 50 mM, YPD Benomyl 500 μg/ml, YPD CuSO_4_ 10 mM, and YPD Sodium meta-arsenite 2.5 mM (Fig. 2A and Supplementary Table S9). Benchmark fitness genes for benomyl, caffeine, copper(II) sulfate, and sodium meta-arsenite were collected from the SGD or manually curated from the literature (see Supplementary Table S10). Manually curated benchmark genes were significantly enriched for YPD Sodium meta-arsenite 2.5 mM using CNVs in the top 1% of genes (Supplementary Table S11). SGD copper benchmark fitness genes were significantly enriched in the top 5% of SNPs from the YPD Sodium meta-arsenite 2.5 mM model (Supplementary Table S11). No benchmark gene enrichment was observed in the top 10% of features for any model (Supplementary Table S11). SGD caffeine benchmark genes were significantly enriched in the top 15% and 20% of SNP features from both the YPD CuSO_4_ 10 mM and YPD Caffeine 50 mM models and in the top 25% of SNP features from the YPD Caffeine 40 and 50 mM models (Supplementary Table S11). These benchmark gene enrichment analyses confirm that predicted genes are functionally relevant to fitness in their respective environment (i.e. caffeine benchmark genes enriched in the YPD Caffeine 50 mM model’s important features).

To further assess how well feature importances aligned with experimentally derived fitness effects, we collected a differential relative fitness dataset of 4165 single mutants under 30 μg/ml benomyl treatment [83]. We expected that the differential relative fitness for 3,101 of these mutants with fitness defects would be correlated with the SHAP values of the corresponding genes from the YPD Benomyl 500 μg/ml model trained on the complete SNP feature set; however, we did not see a significant correlation. While this dataset is extensive, the experiments were performed using a laboratory strain, S288C, that is not included in the population we examined. In addition, their benomyl concentration was 17-fold lower (30 μg/ml). Thus, further experimental study under a similar concentration of benomyl will be crucial to further validate our models.

### Non-benchmark genes explain more fitness variation than benchmark genes

There are four potential reasons for the limited enrichment of environment-specific and cross-environment benchmark fitness genes. First, some of the important genes contributing to predictions may be false positives. While the *R*^2^ was as high as ∼0.7, the models are far from perfect, and false predictions are expected. Second, the fitness trait distributions suggest complex genetic bases (Fig. 2B), and genes with smaller fitness effects are inherently harder to verify experimentally. Thus, it is likely that a substantial number of relevant genes are not present in the benchmark sets. Third, the genetic background in which benchmark fitness genes were experimentally verified was limited to laboratory strains, particularly S288C and W303, the former of which is absent from the diploid budding yeast population dataset. Since laboratory strains have undergone extensive selection under controlled conditions, benchmark genes, which are predominantly discovered in the lab strains, may not all be important for fitness in natural isolates. Fourth, stress-resistance genes are often revealed by generating inactivating mutations such as conditional alleles and/or gene knockouts in laboratory strains. If such mutations lead to substantial fitness defects, they are not likely to occur in natural isolates. Thus, the level of genetic variation in stress-resistance genes may be insufficient for explaining fitness variation, which may make benchmark genes difficult to predict as important.

**Figure 4.**
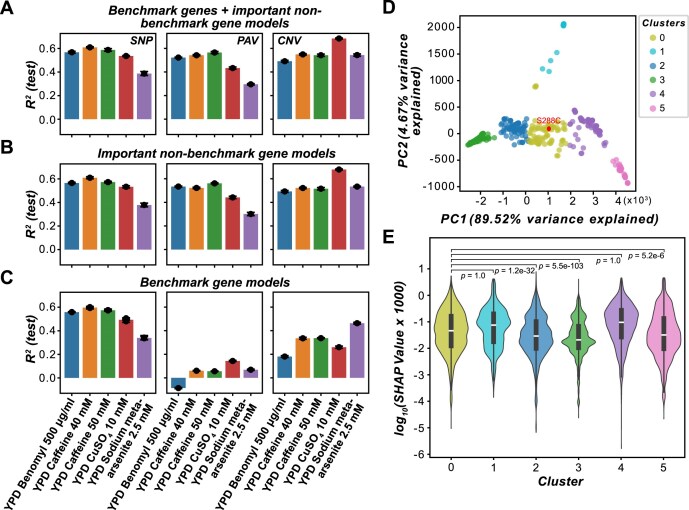
Contribution of benchmark genes to fitness predictions, as well as comparisons of SHAP values between clusters of isolates. (A–C) Performance of RF fitness prediction models built with different feature sets to predict fitness in YPD Benomyl 500 μg/ml, YPD Caffeine 40 mM, YPD Caffeine 50 mM, YPD CuSO_4_ 10 mM, and YPD Sodium meta-arsenite 2.5 mM. Feature sets consisted of (**A**) both important non-benchmark genes identified by the optimized RF models and benchmark genes, (**B**) only important non-benchmark genes, or (**C**) only benchmark genes. Error bars: mean and standard deviation of model performance across 20 evaluation repetitions. (**D**) PC analysis was performed on the Euclidean distance matrix calculated from the SNP genotypes to assess genetic relatedness among isolates. Isolates are colored according to memberships in six K-means clusters of similar isolates identified using the same distance matrix. (**E**) Violin plot of the distributions of the absolute SHAP values from the optimized YPD Caffeine 50 mM SNP model for the clusters of isolates identified in panel (D). *P*-values are from Mann–Whitney U tests conducted to assess the relationship of median absolute SHAP values of benchmark genes between clusters.

If the first possibility is true, we expect that when a model is trained with features devoid of benchmark gene sets, the model performance should decrease appreciably because the predictive power is mainly derived from benchmarks (experimentally verified fitness genes from SGD) in the training data, rather than the important genes we identified that have not been experimentally validated, which we refer to as important non-benchmark genes hereafter. Conversely, if the second possibility is true, i.e. there remains a substantial number of relevant genes not covered by the benchmark genes, then the models devoid of benchmarks would perform just as well as, if not better than, the models including benchmarks. To test this, a new set of RF models were trained using feature sets consisting of only benchmark genes, only important non-benchmark genes, or both benchmark and important non-benchmark genes (see “Materials and Methods” section). Models trained on the feature set consisting of both benchmark and non-benchmark genes (“combined models,” Fig. 4A) or those consisting of only important non-benchmark genes (Fig. 4B) performed better than models built using benchmark genes alone (Fig. 4C) when using PAVs and CNVs. Differences in test *R*^2^ between the important non-benchmark gene models or the combined models and the benchmark gene models ranged from 0.07 to 0.62 and 0.08 to 0.61, respectively. There were less performance differences between the SNP-based models (differences in test *R*^2^ between important non-benchmark gene models or the combined models and the benchmark gene models ranged from 0 to 0.05 and 0.01 to 0.06, respectively, Fig. 4A–C). These results indicate that structural variation in non-benchmark genes explains more variation in fitness than benchmark genes across the selected environments. It is possible that the better or similar performances of important non-benchmark gene-based models are due to more features being used, but there was no significant association between the number of features used for training and model performance (Supplementary Table S12), making this possibility unlikely. Taken together, our results suggest that the limited enrichment of benchmark genes is not likely due to the first possibility (predicted important genes are false positives) and support the second possibility, where the important non-benchmark genes explain a high proportion of variance in fitness.

Next, we reasoned that if the third possibility is true, i.e. benchmarks predominantly discovered in laboratory strains like S288C are less important for fitness in wild yeast isolates, then the feature importance (absolute values of the SHAP values) of benchmark fitness genes should decrease for yeast isolates that are increasingly genetically distant to laboratory strains. To test this, we first clustered the isolates based on their genetic distance, identifying a cluster containing S288C (yellow cluster 0; SNP: Fig. 4D; PAV: Supplementary Fig. S5) and other clusters with varying genetic distances to S288C (non-yellow clusters; Fig. 4D and Supplementary Fig. S5), and then compared the SHAP values from the optimized RF models for benchmark genes between clusters. Using the SNP-based YPD Caffeine 50 mM model as an example, the caffeine benchmark gene SHAP values were significantly greater for isolates more related to S288C (cluster 0) than for the more distantly related isolates in clusters 2 (*P* = 1 × 10^–32^), 3 (*P* = 6 × 10^–103^), and 5 (*P* = 5 × 10^–6^), but not clusters 1 and 4 (*P* = 1, Supplementary Table S13, Fig. 4E). For the PAV-based optimized models, only two or fewer benchmark gene features were found among the important features; thus, no comparisons of SHAP values were made between clusters. The association using the SNP models provides support for the third hypothesis that the bias in the identification of benchmark fitness genes from specific genetic backgrounds, such as laboratory strains, leads to an underestimate of model performance for certain environments. In addition, many genes that were among the important features from the optimized PAV models for the top five best-predicted environments were absent in S288C (mean = 67.7%, sd = 4.8%), partially explaining the lack of enrichment of benchmark fitness genes predicted by PAV models.

To further assess the limited enrichment of benchmark genes among the important genes, we considered that if the fourth hypothesis is true, some benchmark genes are not predicted as important because there is limited genetic variation. To address this, we compared the level of genetic variation between the feature sets of the benchmark genes and the important non-benchmark genes that were used to train the “combined models” in Fig. 4A. We estimated the amount of genetic variation in each dataset by calculating the Euclidean distance between individuals. We found that the distribution of Euclidean distances of isolate pairs based on the benchmark gene features had a significantly lower mean than that based on the important non-benchmark gene features for SNPs, PAVs, and CNVs in three, five, and five environments, respectively (Wilcoxon signed-rank test, *P-*values = 2.2 × 10^–16^, Supplementary Table S12). Low genetic variation can have two forms: (i) either the benchmark genes are present in most of the isolates, or (ii) they are not likely to occur in natural isolates due to their detrimental fitness effects. We found that benchmark genes were present in more isolates compared to the non-benchmark genes on average (Welch’s *t*-test *P*-values: .02 to 7.9 × 10^–18^, Supplementary Table S12). These findings suggest that the apparent lack of importance of benchmark genes in predicting fitness of natural isolates is, in part, due to their importance across all strains, leading to a lack of genetic variation.

### Fitness effects of genetic variants are isolate-dependent

**Figure 5.**
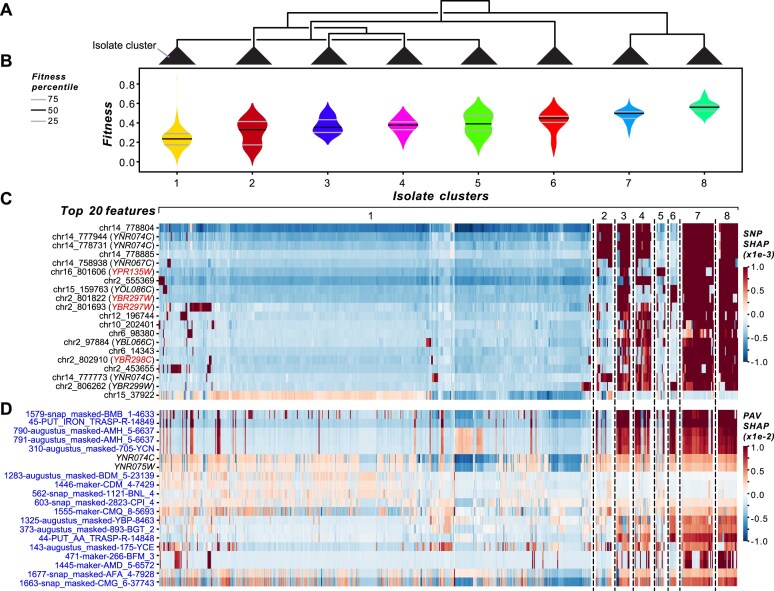
Importance of variants in predicting fitness effects in different isolates. (**A**) Dendrogram showing clusters of isolates based on the SHAP values of the top 20 SNP features from the YPD Benomyl 500 μg/ml optimized model. Dendrogram triangles denote different clusters. (**B**) Violin plot of fitness distributions of isolates in each cluster identified in (A). (**C**) Heatmap of SHAP values of the top 20 SNP features corresponding to the dendrogram in (A). Benchmark benomyl genes are colored in red on the left side of the heatmap. (**D**) Heatmap of SHAP values of the top 20 PAV features. Systematic ORF identifiers are provided for PAV features that mapped to genes. Isolates are ordered based on the SNP-based isolate clusters.

The feature importance analyses reported in earlier sections (e.g. Fig. 3) involve interpreting models globally, i.e. the importance of a variant or a gene indicates its average contribution to fitness predictions among isolates. This raises the question whether the genes that contributed the most to the model predictions do so across most if not all isolates or in an isolate-dependent manner. To address this, we interpreted feature contributions locally, i.e. at the level of individual isolates, using SHAP values from the optimized SNP, PAV, and CNV RF models for the environments with the five best predictions. Positive SHAP values indicate that a genetic variant contributes to an increased predicted fitness, whereas negative SHAP values contribute to a decreased predicted fitness relative to the expected fitness value (the prediction of a model with no features). Isolates were clustered based on the SHAP values of the top 20 most predictive features in each model (see “Materials and methods” section). Clustering of SHAP values across isolates revealed that the feature importances significantly correlated with fitness in 14 out of 15 models, indicating isolate-dependent variant contributions to fitness predictions (SNP: five environments, PAV: five, CNV: four, Supplementary Table S14). For example, SNP-based median absolute SHAP values showed the strongest positive correlation with fitness in YPD Caffeine 40 mM (slope = 306.8, *P *= 1 × 10^–52^, Supplementary Table S14, Supplementary Fig. S6), followed by YPD Benomyl 500 μg/ml (slope = 273.57, *P* = 2 × 10^–42^, Fig. 5A and B, Supplementary Table S14). Similar trends were observed with other variant types, although not as strongly as with SNPs (PAVs: *slope* = −49.2–94.2; CNVs: slope = 34.7–87.0, Supplementary Table S14). We also assessed if the effects of CNVs on fitness predictions are influenced by copy number; we found a significantly positive relationship between CNV values of individual features and fitness in all 35 environments (max *r* among features ranged from 0.2 to 0.6, Supplementary Table S14). Furthermore, this CNV-fitness correlation, as expected, was significantly correlated with the importance of CNV features based on SHAP values for eight environments (adjusted *R*^2^ values ranged from 0.1 to 0.9, *P* ranged from 5.8 × 10^–5^ to 4.9 × 10^–2^). This finding suggests that the contributions of the most important CNVs is associated with higher copy numbers and higher fitness in an isolate.

Next, we focused on YPD Benomyl 500 μg/ml for interpretation because it included benchmark genes among the top 20 most predictive SNP features (Fig. 5C). From the eight clusters identified from the SNP-based SHAP values (Fig. 5A–C), cluster 1 had the lowest median fitness, and most of the top features had negative SHAP values, indicating that these variants contributed to decreased predicted fitness for isolates in cluster 1. In contrast, the same features tended to have positive SHAP values in clusters with higher fitness (clusters 7 and 8). Clusters 2, 3, and 4 showed more heterogeneous SHAP profiles with combinations of both positive and negative contributions that correlated with intermediate to low fitness. Clusters 5 and 6 defy the above generalization but contain a small number of genes with positive SHAP values that are also benchmark benomyl genes (*YPR135W* and *YBR297W*, red feature names, Fig. 5C). *YPR135W* (*CTF4*, Chromosome transmission fidelity) is required for sister chromatid cohesion [95]. Mutations in various *CTF* genes were found to exhibit both tolerant and sensitive growth phenotypes in yeast grown under benomyl stress [96]. *YBR297W* (*MAL33*, Maltose activator) encodes the transcriptional activator that regulates the expression of *MAL31* (maltose permease) and *MAL32* (maltase), which are required for maltose fermentation [97]. Although no direct connection between *MAL31* and benomyl was found, benomyl has been shown to positively affect desirable traits of aneuploid wine-making yeast strains [98]. It would be interesting to investigate the potential effects of benomyl on maltose fermentation of diploid *S. cerevisiae* strains.

The SHAP values of the top 20 PAV (Fig. 5D) and CNV features (Supplementary Fig. S7) generally followed similar clustering patterns as the SNPs but with more admixture. The top 20 PAV and CNV features tended to contribute to lower predicted fitness in isolates from clusters 1 and 2, while a subset of features showed more positive SHAP values across isolates with higher predicted fitness. In clusters 3, 4, 6, 7, and 8, PAV and CNV features generally had more positive SHAP values, but this pattern was inconsistent with the fitness of the SNP-based isolate clusters, where isolates in clusters 3 and 4 tended to have lower fitness and isolates in clusters 6, 7, and 8 tended to have higher fitness. This finding indicates that the PAV and CNV SHAP values do not fully reflect the fitness patterns associated with SNP-based SHAP clusters, highlighting the importance of analyzing multiple variant types to understand the genetic basis of fitness. Furthermore, there was minimal overlap in the genes among the top 20 SNPs and PAVs or CNVs, indicating that each variant type uncovers distinct aspects of the genetic basis of fitness variation across isolates.

We observed that no single feature was overwhelmingly important for predicting fitness in most environments, underscoring the polygenic basis of fitness in these environments (Fig. 5 and Supplementary Figs S6–S10). The most notable exception was the YPD CuSO_4_ 10 mM CNV model, where fitness variation is driven mainly by *CUP1-2* (*YHR055C*, Supplementary Fig. S8), the major gene controlling copper toxicity response in yeast [99]. Another notable exception is an ORF absent in the S288C reference genome (1594-snap_masked-AMH_5-6573) that is a major contributor to higher fitness in the YPD Caffeine 40 mM environment for one cluster of isolates (cluster 8 in Supplementary Fig. S6). The closest BLASTx and tBLASTx match for this ORF (percent identity = 96.93, *E*-value = 0, Supplementary Table S15) is the gene *YLR342W* (*FKS1*, FK506 Sensitivity), which encodes the catalytic subunit of 1,3-β-D-glucan synthase, which is found in the cell wall of most fungi [100]. It remains unclear how this gene may impact response to caffeine. Lastly, in the PAV-based YPD Sodium meta-arsenite 2.5 mM model, *YGL258W* (*VEL1*, Velum formation), a protein with unknown function in budding yeast, contributed to lower predicted fitness in a subset of isolates, whereas the corresponding CNV model identified several genes known to confer tolerance to arsenate when overexpressed [101], including *ARR1* (Arsenicals resistance, *YPR199C*), *ARR2* (*YPR200C*), *ARR3* (*YPR201W*), *YPR196W* (Putative maltose-responsive transcription factor), and *YPR198W* (*SGE1*, Suppression of Gal11 expression), as contributing to higher predicted fitness in certain isolates (Supplementary Fig. S9). Taken together, our findings indicate that model interpretation based on SHAP values allows identification of genetic variants important for predicting fitness in different isolates and hypothesizing which stress-response genes drive fitness differences among isolates. It is also notable that the majority of features within the top 20 are either intergenic SNPs or ORFs not found within the reference genome (S288C), providing an opportunity for further experimental exploration.

### Genetic interactions underlying fitness variation

**Figure 6.**
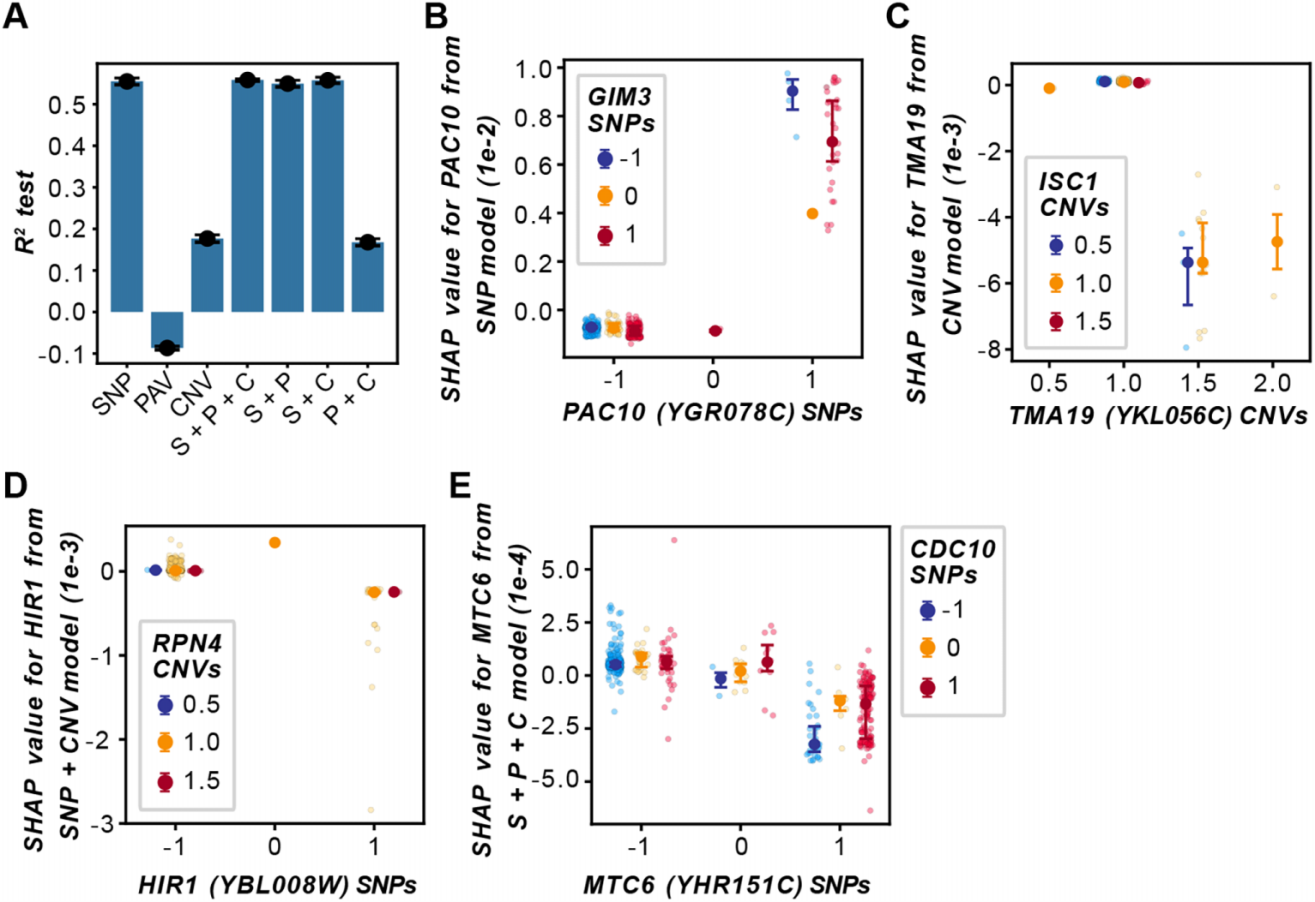
Contribution of genetic interactions to fitness predictions. (**A**) Test set performance *R*^2^ values for the prediction of fitness in YPD Benomyl 500 μg/ml by RF models trained on the benomyl benchmark genes. SNP, PAV, and CNV variants were mapped to genes (see “Materials and methods” section), and one variant with the highest feature importance was selected to represent the gene in the model. Models were trained on individual SNP, PAV, and CNV datasets or integrated datasets (S: SNP, P: PAV, C: CNV). Feature interactions between (**B**) *PAC10* SNP and *GIM3* SNP, (**C**) *TMA19* CNV and *ISC1* CNV, (**D**) *HIR1* SNP and *RPN4* CNV, and (**E**) *MTC6* SNP and *CDC10* SNP. Axes: SNP or CNV genotypes of a gene (*x*-axis) and the gene’s corresponding SHAP values (*y*-axis). SNP genotypes are encoded as −1 (homozygous for the major allele), 0 (heterozygous), or 1 (homozygous for the minor allele). Points represent isolates and the color represents the SNP, PAV, or CNV genotypes of the second gene. Points with error bars represent the median SHAP value and the interquartile range at the 25th and 75th percentiles.

We found that multiple important features that are predictive of fitness in an environment show similar SHAP value patterns within the same genetic backgrounds (Fig. 5C and D; Supplementary Figs S6–S10), indicating that they contribute similarly to fitness predictions, potentially via shared genetic mechanisms or genetic interactions. To assess the contribution of genetic interactions to fitness predictions, we first asked if experimentally validated genetic interactions are predictive of fitness. To do this, we used RF-based models, which can better capture non-linear feature interactions (i.e. gene–gene interactions) [78, 102] than linear models, to obtain predicted genetic interactions using SHAP [78]. Models were trained with either individual or combinations of genetic variant types to identify gene–gene interactions. We focused on YPD Benomyl 500 µg/ml because experimentally validated genetic interaction data are available for this environment. Models were trained using only benchmark benomyl genes from SGD (Supplementary Table S10). To assess the biological relevance of SHAP-based predictions, we compared them with experimentally validated genetic interaction networks from the BioGRID database and two additional publicly available networks: for benomyl 30 μg/ml [83] and a control condition [83] (Supplementary Fig. S11). The SNP model performed similarly to the combined SNP + PAV + CNV, SNP + PAV, and SNP + CNV RF models (all models: testing *R*^2^ ≈ 0.55, Fig. 6A). In contrast, combining PAVs and CNVs improved performance (testing *R*^2^ = 0.17) compared with PAVs alone (testing *R*^2^ = −0.09), but not over CNVs alone (testing *R*^2^ = 0.18). Model performances were not significantly associated with the number of features used to train the models (Supplementary Table S16). These results suggest that PAVs and CNVs of benomyl benchmark genes may not be as informative as SNPs in explaining fitness in the YPD Benomyl 500 µg/ml environment and that SNPs may be able to identify more relevant genetic interactions than other variant types in this environment.

We identified a total of 210 758 feature interactions from the YPD Benomyl 500 µg/ml RF models in Fig. 6A, excluding the PAV model because of its negative performance (Supplementary Table S17); of these 69 486 represented unique gene–gene interactions. We reasoned that if these gene–gene interactions are biologically meaningful, they may be identified by multiple genetic variant types. To examine this, we determined which types of variant–variant feature interactions mapped to the same gene pairs (Supplementary Table S18), and whether those gene pairs have been experimentally validated according to the literature. Consistent with our hypothesis, there was substantial overlap in the gene–gene interactions identified by different variant combinations (Supplementary Fig. S12). In addition, although only one of the unique gene–gene interactions overlapped with the Benomyl 30 μg/ml network, 59 overlapped with the control condition network and 6358 overlapped with BioGRID interactions. Of the 6358 validated genetic interactions, 5645 exhibited feature interactions for two or more variant–variant pair types (Supplementary Table S18), with SNP–SNP interactions identifying the most validated genetic interactions (6095 out of 6358 gene pairs). Similar to what we discovered for the benchmark genes, these experimentally validated genetic interactions were not significantly enriched in the SHAP-based feature interactions we identified (*q *> 0.38, Supplementary Table S19) regardless of the rank percentile examined (see “Materials and Methods” section).

One potential reason for the lack of enrichment is limited coverage of the predicted interactions, as these were derived from models based on SGD benchmark genes with sensitive phenotypes under 500 μg/ml benomyl. Thus, we re-estimated the SHAP interaction values using models built with single variant types or combinations of variant types, including both important non-experimentally validated genes and genes from the SGD benchmark set. We found that, similar to what we found with single gene analysis—where SHAP values were correlated with differential relative fitness—there was low to no significant correlation between genetic interaction scores from the published dataset [83] and SHAP interaction values for different isolates. Nonetheless, the integrated SNP + PAV + CNV model recovered the most experimentally validated genetic interactions (400 gene pairs from the Benomyl 30 μg/ml network with significant genetic interaction scores: *P *< .05), highlighting the importance of considering multiple genetic variant types to better understand the contributions of genetic interactions to fitness variation. Similar to the single gene analysis, it is possible that the lack of correlation between predicted and experimental scores (fitness effects and genetic interaction strengths) is due to differences between the datasets used for model training and experimental validation. Thus, further experimental validation with a higher concentration of benomyl is warranted.

However, the SHAP-based interactions may represent novel, biologically relevant genetic interactions that are good candidates for experimental validation. Furthermore, SHAP-based feature interactions provide insight into potential genetic mechanisms affecting the ability of interacting genes to contribute to fitness predictions. For example, the single Benomyl 30 μg/ml genetic interaction was between *PAC10* (Perish in the Absence of Cin8p, *YGR078C*) and *GIM3* (Gene Involved in Microtubule biogenesis, *YNL153C*, ranked 21, Supplementary Table S17). Both genes encode components of the prefoldin co-chaperone complex, which promotes α- and γ-tubulin formation [103]. The minor allele of the *PAC10* SNP contributes to higher predicted fitness when it is homozygous and the *GIM3* SNP is heterozygous, and even higher predicted fitness when the *GIM3* SNP is homozygous for either the major or minor allele (Fig. 6B).

We next examined the top five feature interactions with the highest SHAP interaction values from each model (excluding the PAV model). Of these 30 interactions, five were CNV–CNV interactions from the CNV model involving *TMA19* (Translation machinery associated, *YKL056C*). *TMA19* interacted with *ACL1* (Ankyrin repeat chaperone of Rpl1p, *YCR051W*, ranked 1st), *YCL001W-A (*an uncharacterized ORF, ranked 2nd), *IMP21* (Independent of mitochondrial particle, *YIL154C*, ranked 3rd), *ISC1* (Inositol phosphosphingolipid phospholipase C, *YER019W*, ranked 4th), and *YCR025C* (an uncharacterized ORF, ranked 5th, Supplementary Table S17). Although no genetic or physical interactions have been reported in the literature between these genes, the feature interaction between *TMA19* and *ISC1* makes biological sense given the functions of Tma19p and Isc1p. Tma19p translocates to the outer membrane of the mitochondria under stress conditions, including benomyl treatment, as an anti-apoptotic measure [104] and binds with microtubules to stabilize them [104]. Isc1p also translocates to the mitochondria [105], modulates apoptosis by generating bioactive ceramide molecules [106], and is implicated in spindle elongation [107]. The involvement of these genes in apoptosis and cell division may suggest genetic interactions or functional associations, and they are good targets for further experimental validation. Another interesting finding is the association between the copy numbers of *TMA19* and those of *ISC1*. For example, when specific isolates have one copy of *ISC1* and 1.5 (i.e. a full copy and an additional partial copy of the full length of the ORF) or 2 copies of *TMA19*, the *TMA19* CNV tends to contribute to lower predicted fitness (Fig. 6C).

Among the five strongest feature interactions in the SNP + CNV model, two were SNP–CNV interactions of *HIR1* (Histone regulation, *YBL008W*). *HIR1* interacted with *SER1* (3-Phosphoserine aminotransferase, *YOR184W*, ranked 1st) and *RPN4* (Regulatory particle non-ATPase, *YDL020C*, ranked 3rd), the latter of which has been reported by multiple high-throughput studies to negatively interact with *HIR1* [108–110]. Here we found that the contribution of the *HIR1* SNP to fitness predictions depends on the *RPN4* copy number in certain isolates. In isolates with a single copy or partial duplication of *RPN4* and a homozygous SNP genotype for the minor allele of *HIR1*, the *HIR1* SNP contributed to lower predicted fitness (Fig. 6D).

Of the top 30 feature interactions, 14 were SNP–SNP interactions from the SNP, SNP + PAV, and SNP + PAV + CNV models. In the SNP + PAV + CNV model, SNP–SNP interactions often involved *CDC10* (Cell division cycle septin, *YCR002C*), which interacted with *JAC1* (J-type accessory chaperone, *YGL018C*, ranked 1st), *MTC6* (Maintenance of telomere capping, *YHR151C*, ranked 2nd), and *ISC1* (ranked 4th). Costanzo *et al*. [111] confirmed a negative genetic interaction between *MTC2*, another protein involved in maintenance of telomere capping, and *CDC10* under standard growth conditions; thus, *MTC6* may also genetically interact with *CDC10*. Examining the *MTC6* and *CDC10* feature interaction showed that regardless of the genotype of the *CDC10* SNP, the *MTC6* SNP contributes to reduced predicted fitness when isolates are homozygous for the minor allele and to higher predicted fitness when isolates are homozygous for the major allele (Fig. 6E).

Overall, SHAP interaction values are useful for identifying gene pairs that are jointly important for predicting fitness. While enrichment of experimentally validated genetic interactions was not observed, several high-ranking feature interactions involved genes with reported genetic interactions, underscoring the value of utilizing SHAP interaction values for identifying candidate genes for functional validation. The lack of enrichment of experimentally validated genetic interactions may reflect the dependence of genetic interactions on the genetic background and the environment [83]. For example, a subset of experimentally validated genetic interactions were obtained under a less severe benomyl stress (30 μg/ml) than the condition in which the isolates were grown (benomyl 500 μg/ml and YPD media). Furthermore, the experimentally validated genetic interactions also involved non-benomyl benchmark genes, which may also partially explain the lack of enrichment. In addition, several of the top 30 SHAP interactions were observed among genes with related functions, which may be indicative of genetic interactions [83, 111, 112], but further experimental validation is required for confirmation.

## Conclusion

Our study demonstrates that the type of genetic variant—SNP, PAV, or CNV—has a significant impact on both the accuracy of fitness predictions and the biological interpretability of predictive models. Furthermore, structural variants, particularly CNVs, contributed more strongly to predictive performance in most environments and were effective in identifying both known benchmark genes and novel candidate genes involved in environmental stress responses in the environments we assessed. One reason for this is the substantial effects that structural variants such as CNVs can have on gene expression and protein abundance, which in turn lead to fitness variation [62, 88]. For example, CNVs may decrease fitness when they are associated with increased abundance of protein, leading to elevated intracellular solute concentrations, which causes hypo-osmotic stress [62, 113]. On the other hand, CNVs can provide a temporary fitness advantage if they result in increased expression of a gene required for survival in an environment, but decrease fitness when the stress is removed [62]. Furthermore, the number of CNVs may differ from that of SNPs or other variants depending on the genetic backgrounds or fungal species investigated [62, 88]. Thus, future studies of the genotype–phenotype relationship should strongly consider leveraging CNVs to better understand phenotypic variation and adaptation.

Importantly, our models were able to recover key benchmark genes in multiple environments—caffeine, benomyl, CuSO_4_, and sodium meta-arsenite—and candidate non-benchmark genes driving improvements in model performance, suggesting that non-benchmark genes explain a substantial portion of fitness variation. We also found that the genetic background of isolates influenced how individual genes contributed to fitness, with background-dependent effects being driven by many genes instead of a few high-impact genes for five out of 35 environments. Further studies on the effect of genetic background on fitness variation and how the environment alters the contributions of genotypes to fitness are needed to disentangle this complicated relationship.

The data used in this study came from a population of isolates from both human-associated environments, such as wine/sake and beer production, and natural environments such as fruit and soil. There has likely already been selection against genetic variants associated with low fitness in multiple environments. For the growth conditions that are associated with the ecological origins of the isolates, selection has potentially acted against deleterious genetic variants. Thus, the genetic determinants we have identified are likely a mixture of products due to natural as well as artificial selection that need to be further experimentally verified. Overall, our results highlight the complex interplay between genetic variation, environment, and genetic background in shaping fitness. By identifying both shared and environment-specific candidate genes, this study provides insights into the genetic basis of fitness variation in different environments and a foundation for future functional validation experiments. The comparative analysis of benchmark and candidate genes across environments using SHAP values reveals common and distinct mechanisms of fitness, while clustering of isolate-level gene contributions uncovers patterns of coordinated or opposing effects across genetic backgrounds. Together, these findings offer a framework for guiding gene target selection in engineering yeast strains with improved stress resilience.

## Supplementary Material

lqag084_Supplemental_Files

## Data Availability

All data and code needed to reproduce the results from this study are available on Zenodo (supplementary files: https://doi.org/10.5281/zenodo.20027585, code: https://doi.org/10.5281/zenodo.19827560).
