# Supplementary material for: Predictive models of the genetic bases underlying budding yeast fitness in multiple environments
Source: NAR Genom Bioinform. 2026 Aug 11;8(3):lqag084. doi: 10.1093/nargab/lqag084 (PMC13458404; doi:10.1093/nargab/lqag084)
Supplement: Supplementary file 1 [file lqag084_supplemental_files.zip › Revised_supplements_nar_genomics_bioinformatics_v2.pdf]

Supplementary information for:

Predictive models of the genetic bases underlying budding yeast fitness in multiple environments

Kenia E. Segura Abá, Paulo Izquierdo, Gustavo de Los Campos, Melissa D. Lehti-Shiu, Shin-Han Shiu

This document includes:

- Supplementary Figures and Legends S1 to S12
- Supplementary Table Legends S1 to S19
- Supplementary File Legends S1 to S16

Supplemental figure legends

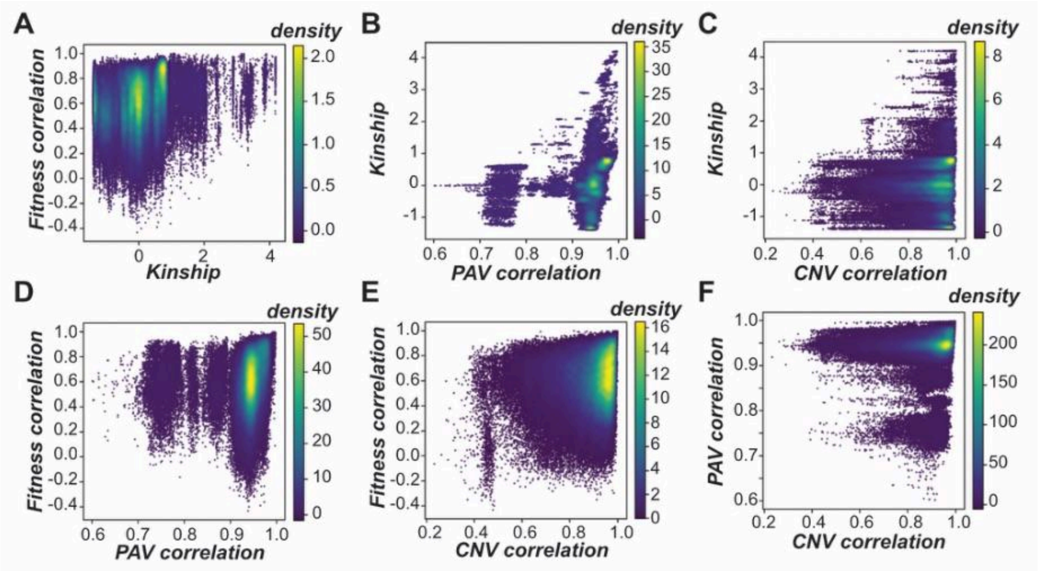

S1 Fig. Correlations between fitness and genetic variant types and relationships between pairs of genetic variant types.

Pearson correlation (r) was calculated to compare the relationships between data type correlation matrices, which were calculated between pairs of isolates. **(A-F)** Density scatter plots showing the relationship between fitness correlation and kinship ($r = 0.23$, $P < 2.2\text{x}10^{-16}$) **(A)**, kinship and PAV correlation ($r = 0.20$, $P < 2.2\text{x}10^{-16}$) **(B)**, kinship and CNV correlation ($r = 0.09$, $P < 2.2\text{x}10^{-16}$) **(C)**, fitness correlation and PAV correlation ($r = 0.15$, $P < 2.2\text{x}10^{-16}$) **(D)**, fitness correlation and CNV correlation ($r = 0.13$, $P < 2.2\text{x}10^{-16}$) **(E)**, and PAV correlation and CNV correlation ($r = 0.06$, $P < 2.2\text{x}10^{-16}$) **(F)**.

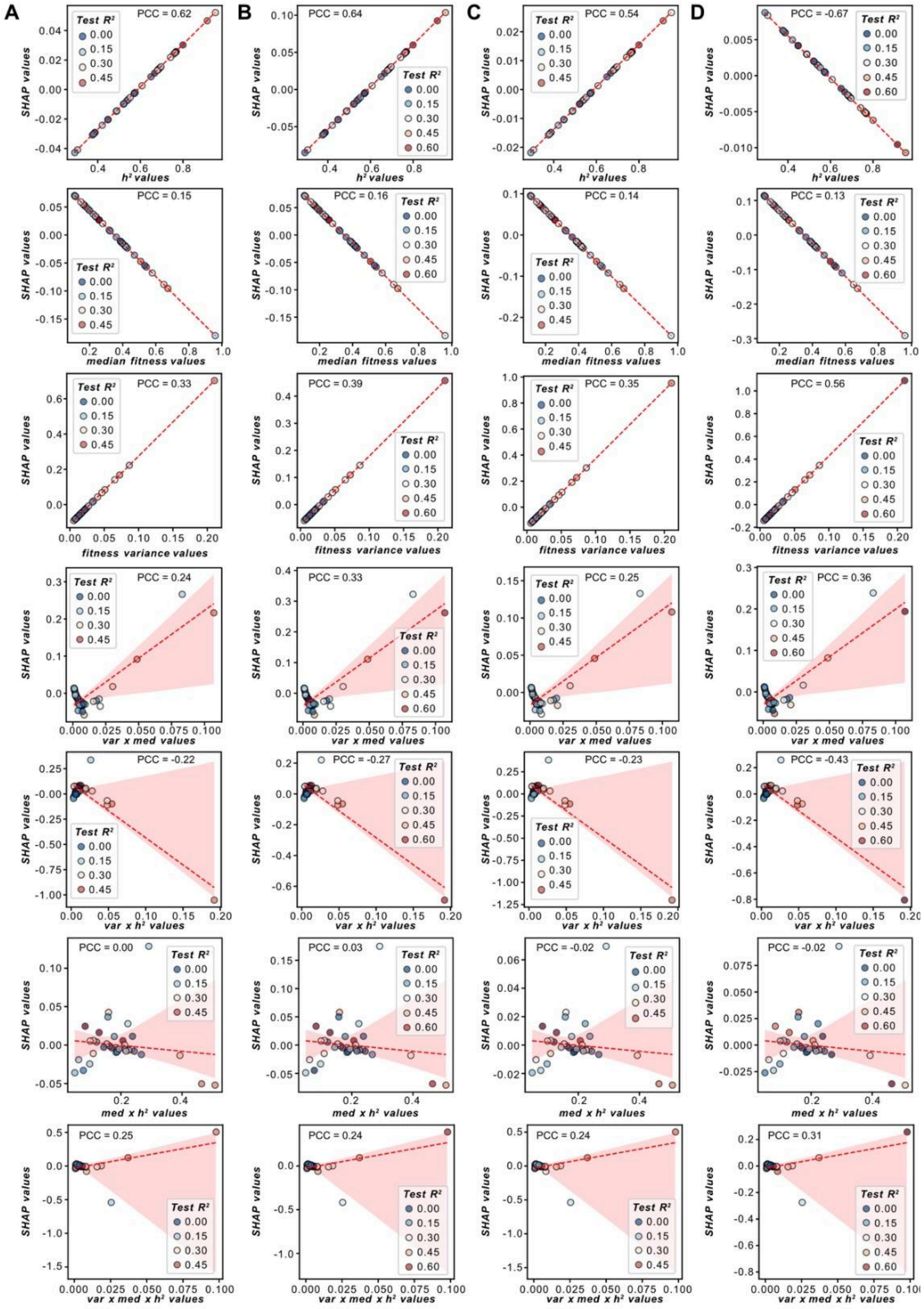

S2 Fig. SHAP values of fitness-related features

(A-D) Scatterplots showing the relationship between the values of a fitness-related feature (x-axis, i.e., h^2, median fitness, fitness variance, and their pairwise and three-way interactions) and the SHAP

values of that feature (y-axis) from each **(A)** PC-, **(B)** SNP-, **(C)** PAV-, and **(D)** CNV-based linear model that was built to predict single-environment optimized RF model performances. Points are colored by the testing set R^2 performance. The Pearson correlation values between the SHAP values of a feature versus the testing set R^2 are shown.

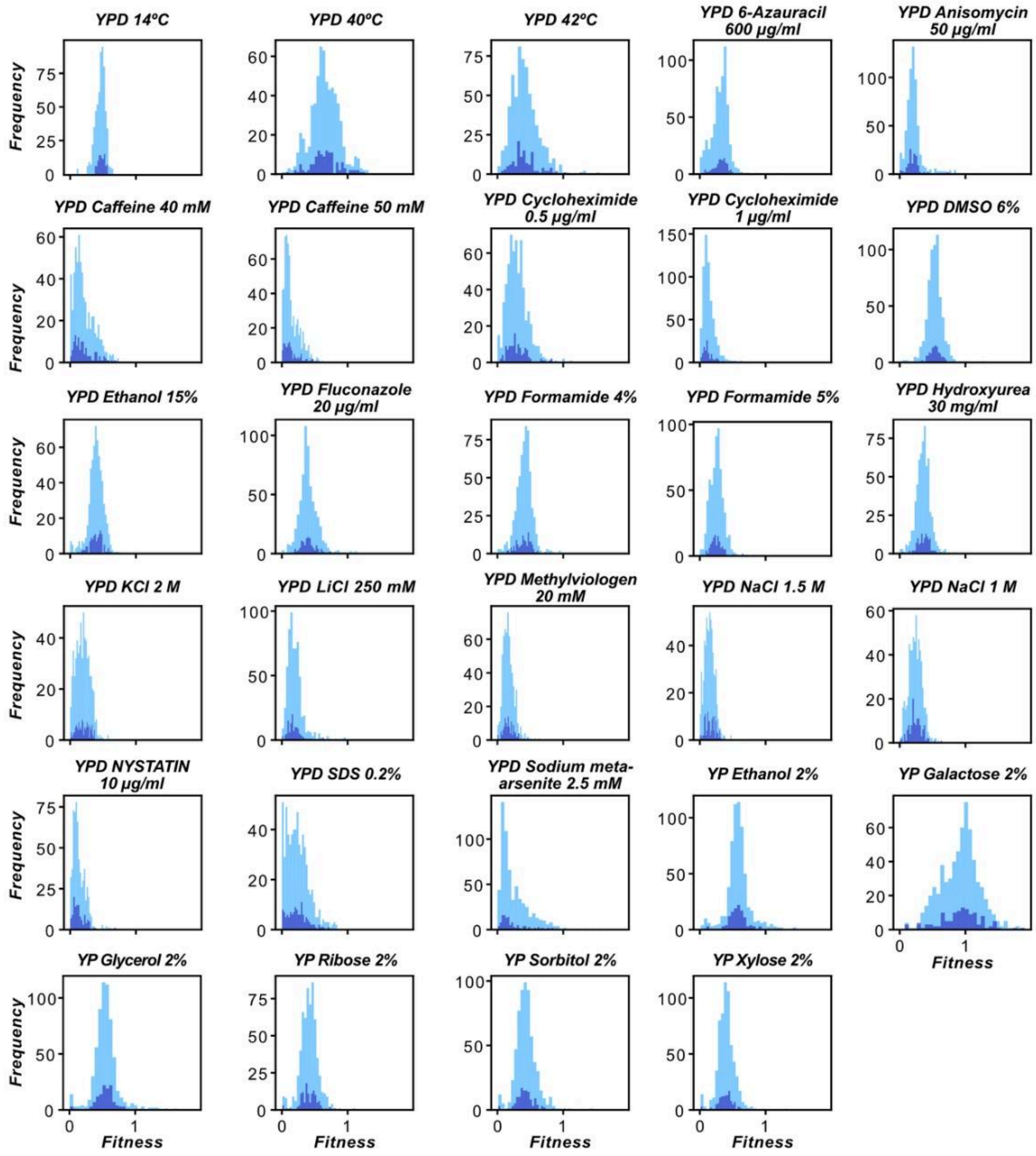

S3 Fig. Fitness distributions of isolates in each environment.

Fitness distributions of the 625 diploid isolates used for training models (blue) and the 125 isolates (purple) used for testing are shown for 29 environments.

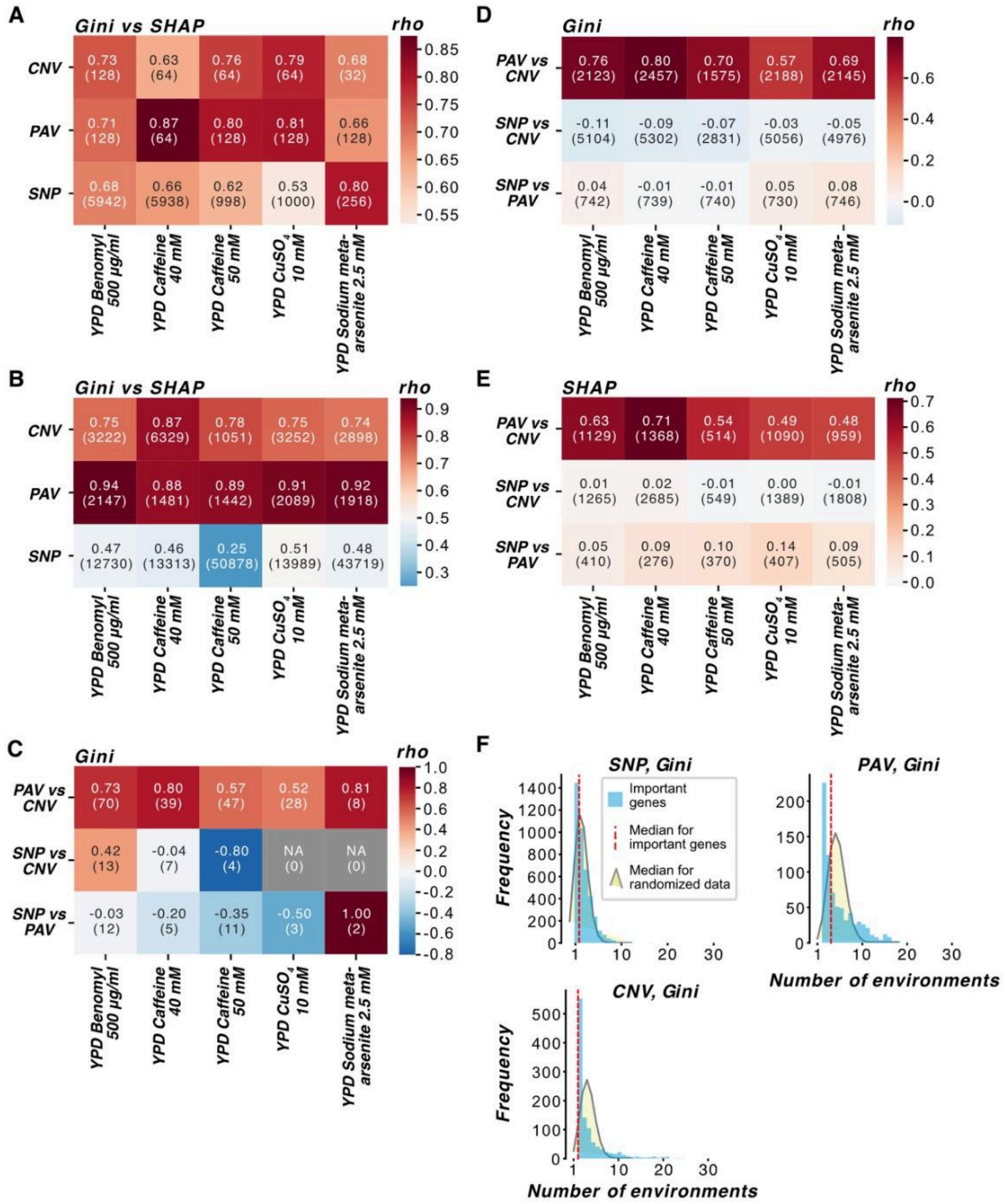

S4 Fig. Relationships between Gini and SHAP feature importance in different environments. **(A-B)** Spearman's rank correlation between feature rankings based on Gini importance and average SHAP values for **(A)** the optimized single-environment RF models and **(B)** single-environment RF models trained on complete SNP, PAV, or CNV feature sets. The number of overlapping features are shown in the parenthesis. **(C-E)** Spearman's rank correlations between genetic variant types using Gini feature importance from the **(C)** optimized RF models and **(D)** the RF models built using complete feature sets and **(E)** using average SHAP values for the RF models trained on complete

feature sets. The number of overlapping genes are shown in the parenthesis. See **Materials and Methods** for details about the gene to feature mappings. **(F)** Gini importance values from the optimized RF models were used to determine the distribution of unique or shared genes with non-zero importance across 1 to 35 environments (blue bars; red line: median). This distribution was compared to a null distribution of median randomized counts (see **Materials and Methods**) using the Kolmogorov-Smirnov test (alternative = "greater"); for SNPs: median $P = 5.9 \times 10^{-28}$; for PAVs: $P = 1.5 \times 10^{-31}$; for CNVs: $P = 4.7 \times 10^{-71}$.

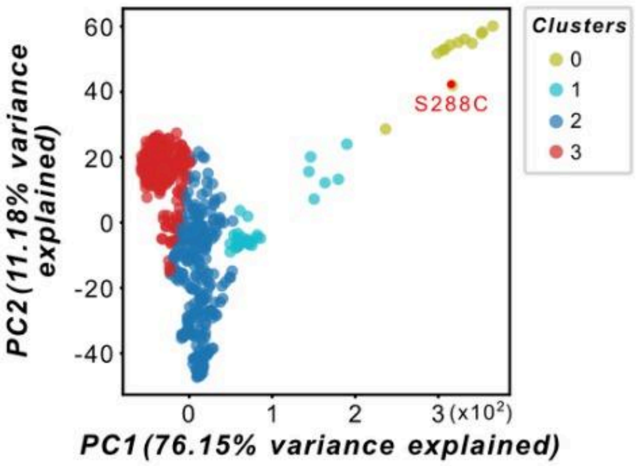

S5 Fig. Genetic relatedness of isolates to laboratory strains based on PAVs.

Principal component analysis of the Euclidean distance matrix of the PAV genotypes was performed to assess genetic relatedness among isolates. Clusters of genetically similar isolates were identified using K-means clustering applied to the distance matrix. Four clusters were identified from the PAV matrix. S288C is labeled in the plot.

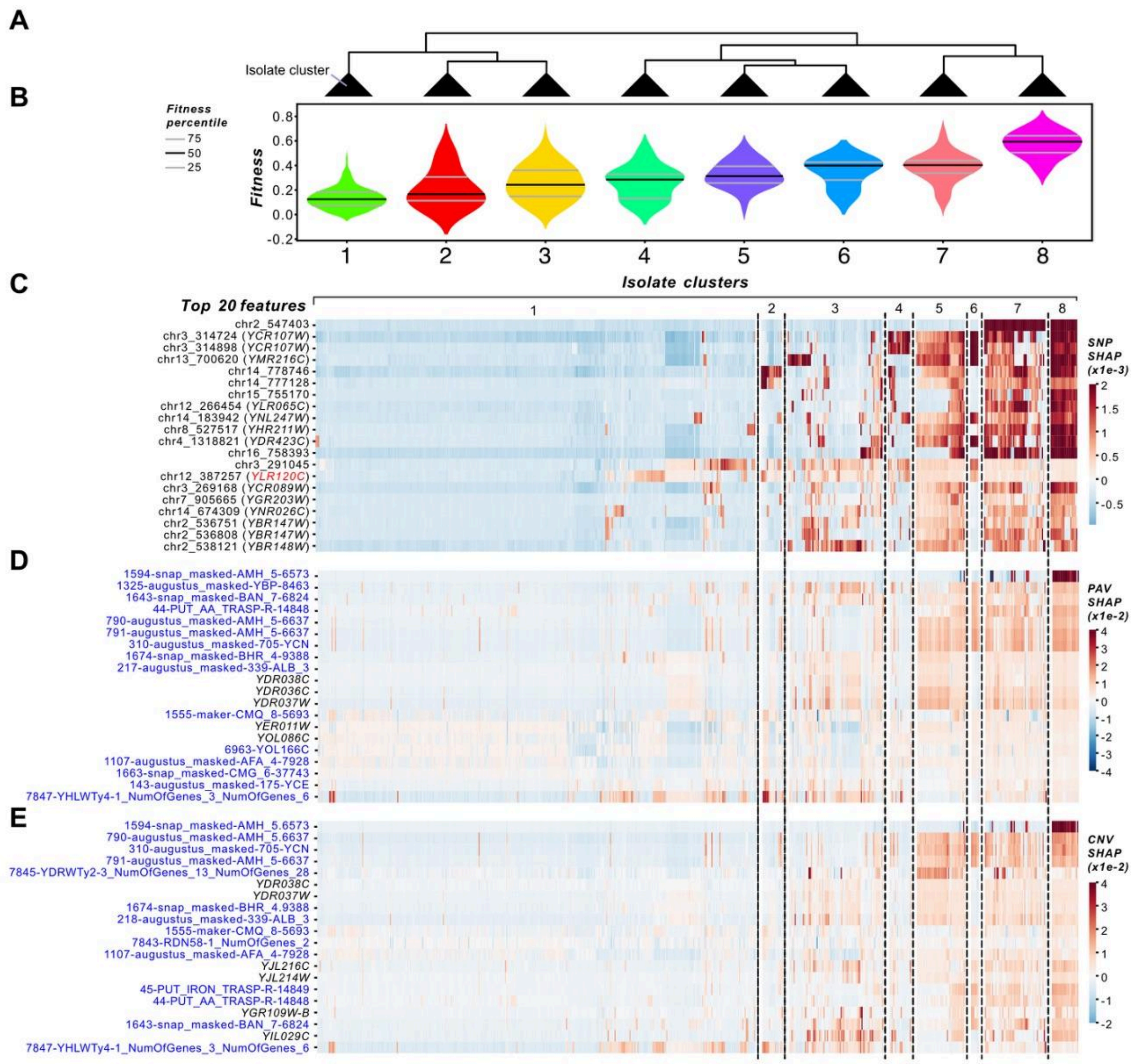

S6 Fig. Isolate-dependent effects of SNP, PAV, and CNV features on fitness in YPD Caffeine 40 mM.

(A) Dendrogram showing clusters of isolates based on the SHAP values of the top 20 features from the YPD Caffeine 40 mM optimized SNP model. **(B)** Violin plot of fitness distributions of isolates in each cluster identified in (A). Heatmap of SHAP values of the top 20 **(C)** SNP, **(D)** PAV, and **(E)** CNV features from the optimized YPD Caffeine 40 mM models. ORF features are colored in blue and features that are mapped to genes are colored in black on the left side of the heatmaps. Heatmap cells are colored by SHAP value. Isolates are ordered based on the SNP-based isolate clusters.

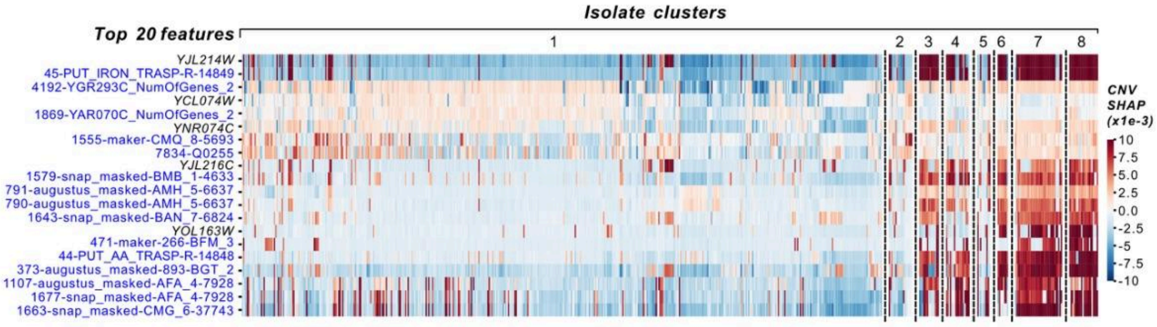

S7 Fig. Isolate-based clusters of CNV feature SHAP values from the optimized YPD Benomyl 500 µg/ml RF model.

Heatmap of SHAP values of the top 20 CNV features from the optimized YPD Benomyl 500 µg/ml RF model. ORF features are colored in blue and features that are mapped to genes are colored in black on the left side of the heatmap. Heatmap cells are colored by SHAP value and isolates are ordered based on the SNP-based isolate clusters.

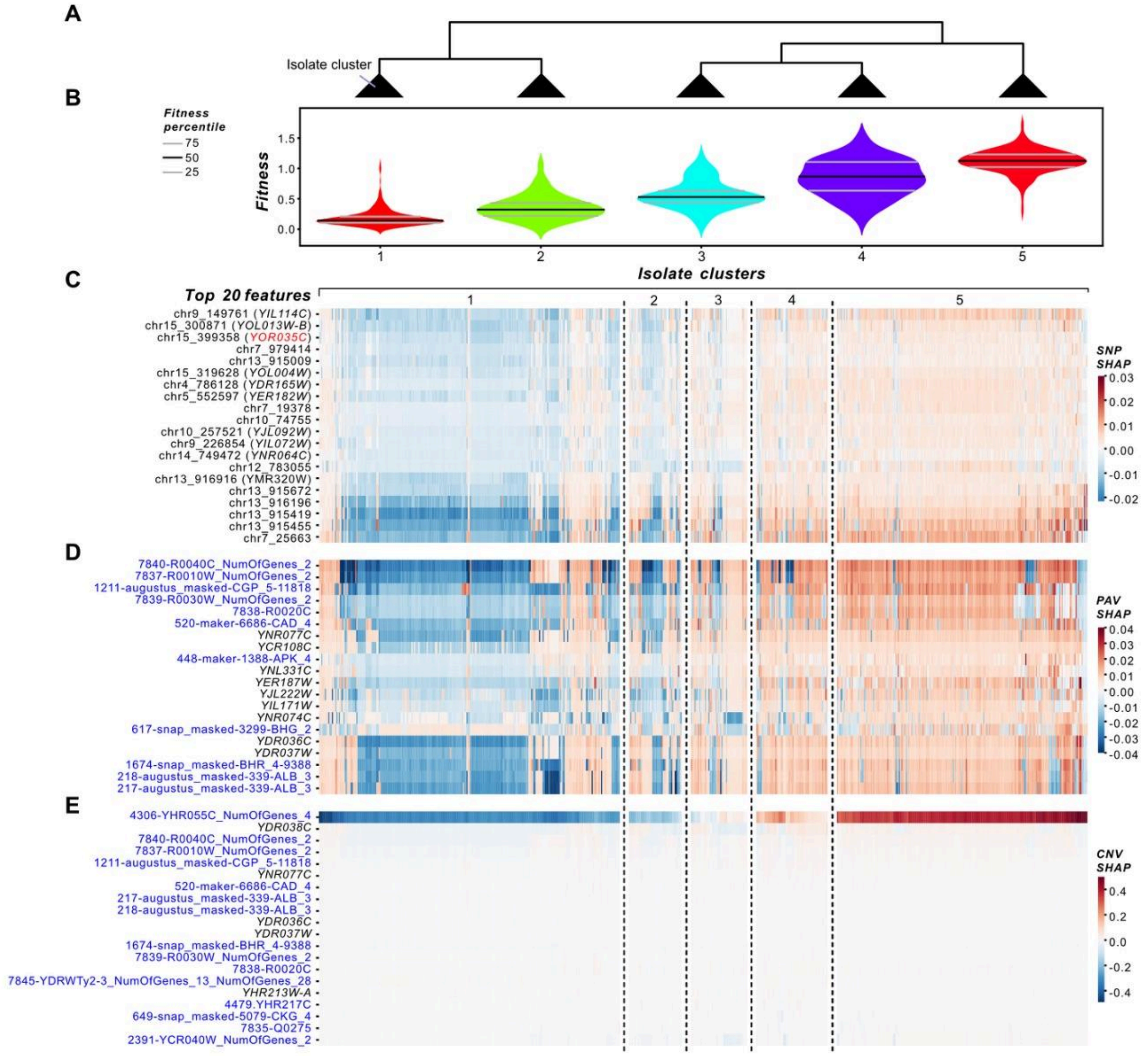

S8 Fig. Isolate-dependent effects of SNP, PAV, and CNV features on fitness in YPD $CuSO_4$ 10 mM.

(A) Dendrogram showing clusters of isolates based on the SHAP values of the top 20 features from the YPD $CuSO_4$ 10 mM optimized CNV model. **(B)** Violin plot of fitness distributions of isolates in each cluster identified in (A). Heatmap of SHAP values of the top 20 **(C)** SNP, **(D)** PAV, and **(E)** CNV features from the optimized YPD $CuSO_4$ 10 mM models. ORF features are colored in blue and features that are mapped to genes are colored in black on the left side of the heatmaps. Heatmap cells are colored by SHAP value. Isolates are ordered based on the CNV-based isolate clusters.

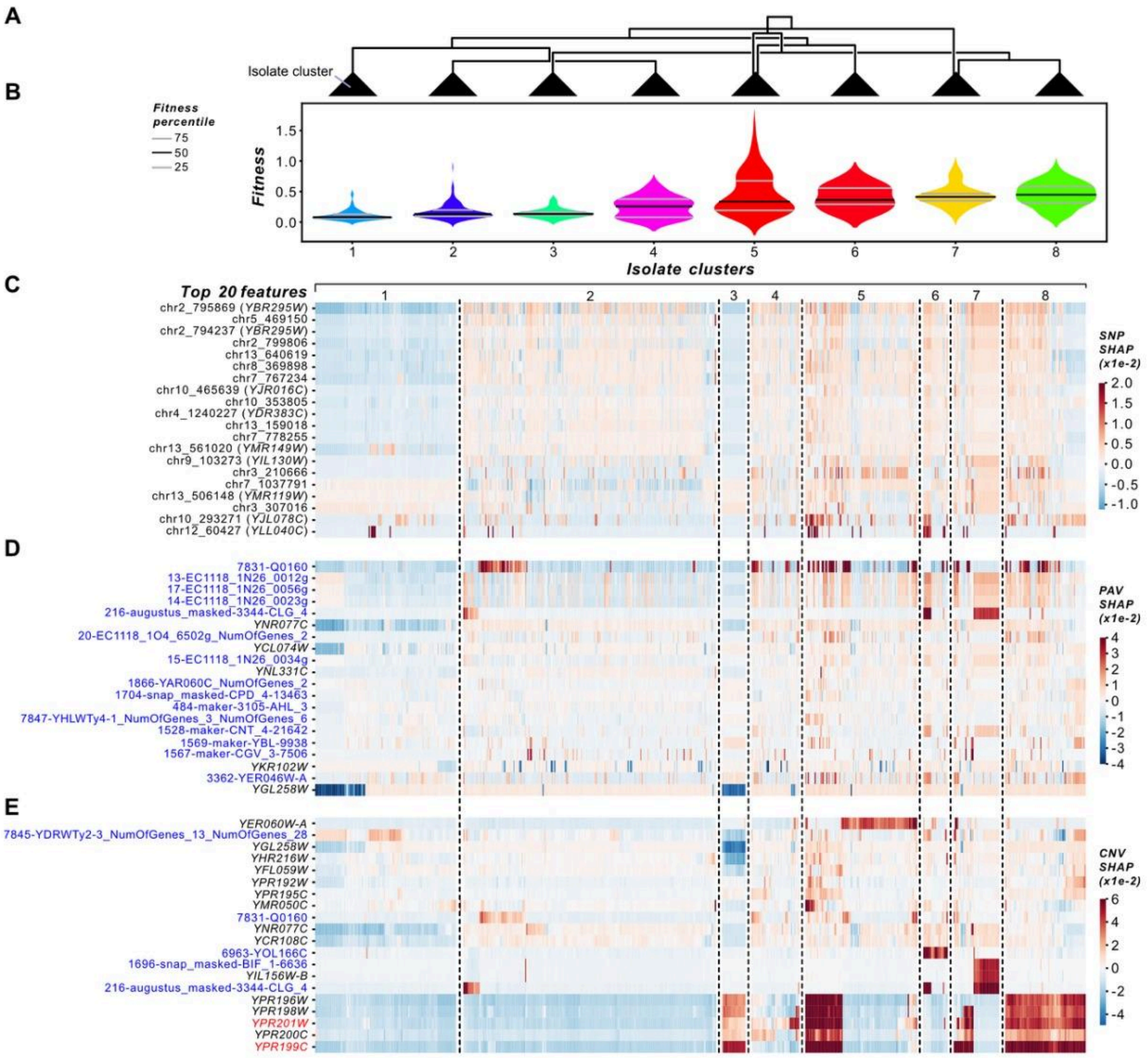

S9 Fig. Isolate-dependent effects of SNP, PAV, and CNV features on fitness in YPD Sodium meta-arsenite 2.5 mM.

(A) Dendrogram showing clusters of isolates based on the SHAP values of the top 20 features from the YPD Sodium meta-arsenite 2.5 mM optimized CNV model. **(B)** Violin plot of fitness distributions of isolates in each cluster identified in (A). Heatmap of SHAP values of the top 20 **(C)** SNP, **(D)** PAV, and **(E)** CNV features from the optimized YPD Sodium meta-arsenite 2.5 mM models. Benchmark gene systematic identifiers are colored in red, ORF features are colored in blue, and features that are mapped to genes are colored in black on the left side of the heatmaps. Heatmap cells are colored by SHAP value. Isolates are ordered based on the CNV-based isolate clusters.

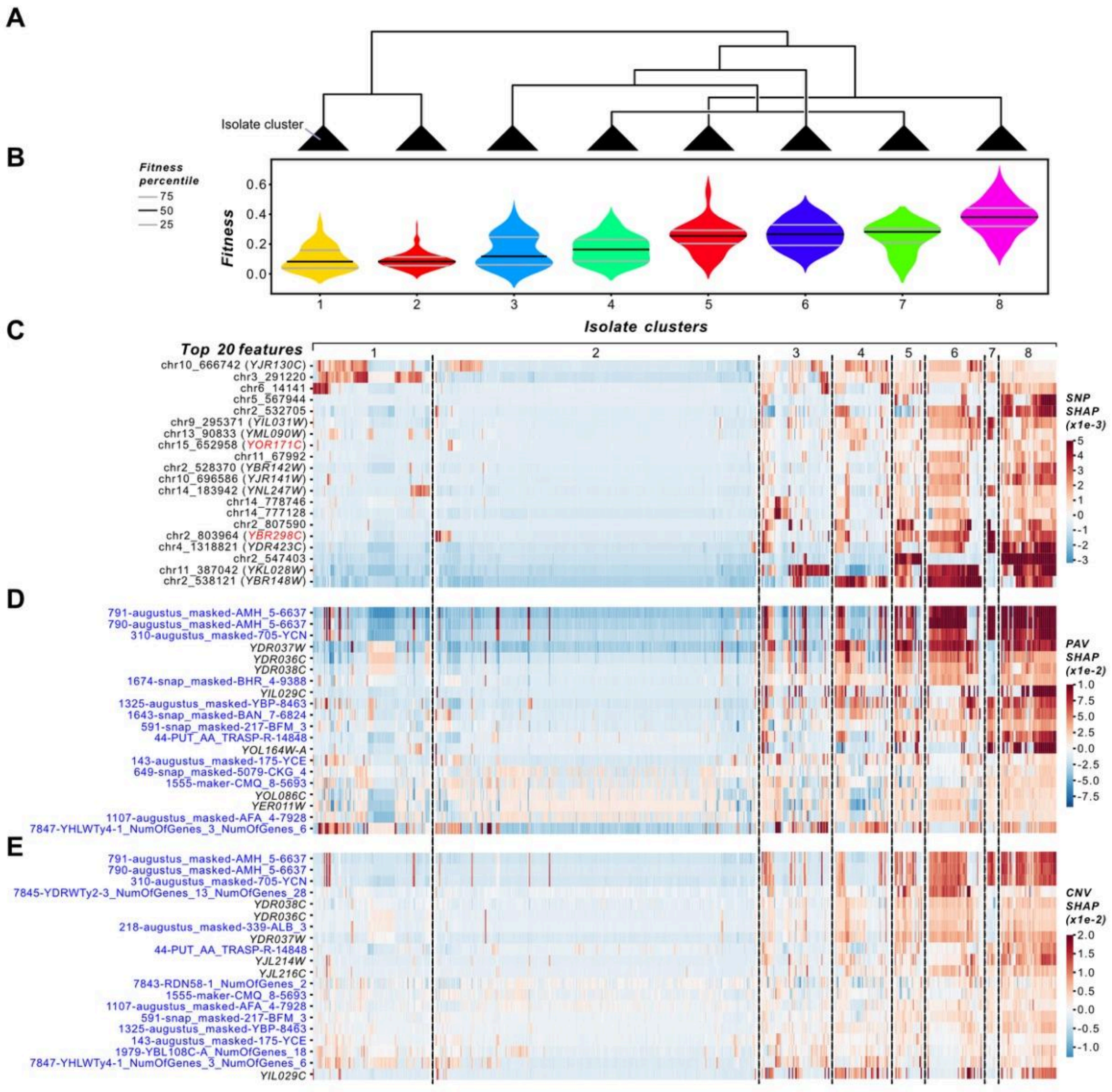

S10 Fig. Isolate-dependent effects of SNP, PAV, and CNV features on fitness in YPD Caffeine 50 mM.

(A) Dendrogram showing clusters of isolates based on the SHAP values of the top 20 features from the YPD Caffeine 50 mM optimized SNP model. **(B)** Violin plot of fitness distributions of isolates in each cluster identified in (A). Heatmap of SHAP values of the top 20 **(C)** SNP, **(D)** PAV, and **(E)** CNV features from the optimized YPD Caffeine 50 mM models. Benchmark gene systematic identifiers are colored in red, ORF features are colored in blue, and features that are mapped to genes are colored in black on the left side of the heatmaps. Heatmap cells are colored by SHAP value. Isolates are ordered based on the SNP-based isolate clusters.

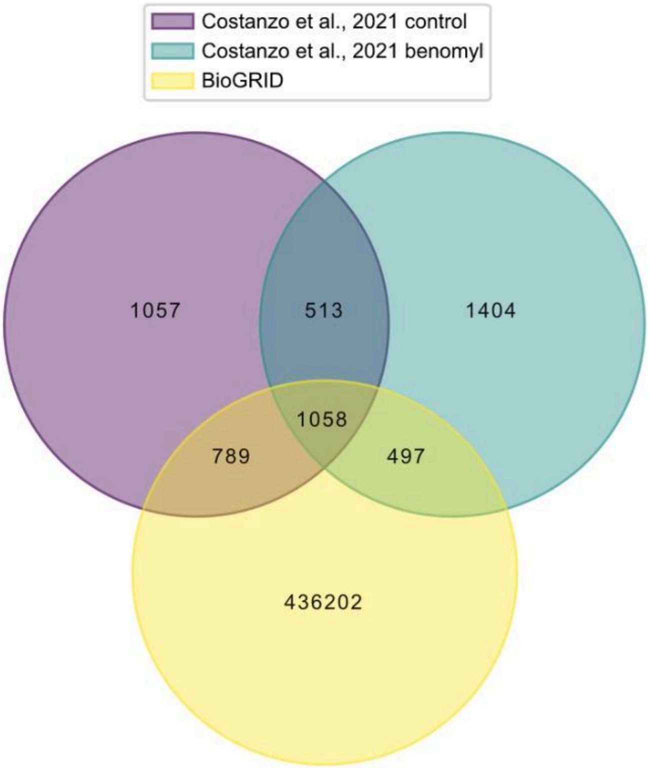

S11 Fig. Experimentally validated genetic interactions from BioGRID and Costanzo et al., 2021. Venn diagram showing the overlap in genetic interactions collected from BioGRID (yellow) and genetic interactions validated by Costanzo et al., 2021 under the control condition (purple) and under 30 µg/ml benomyl (blue).

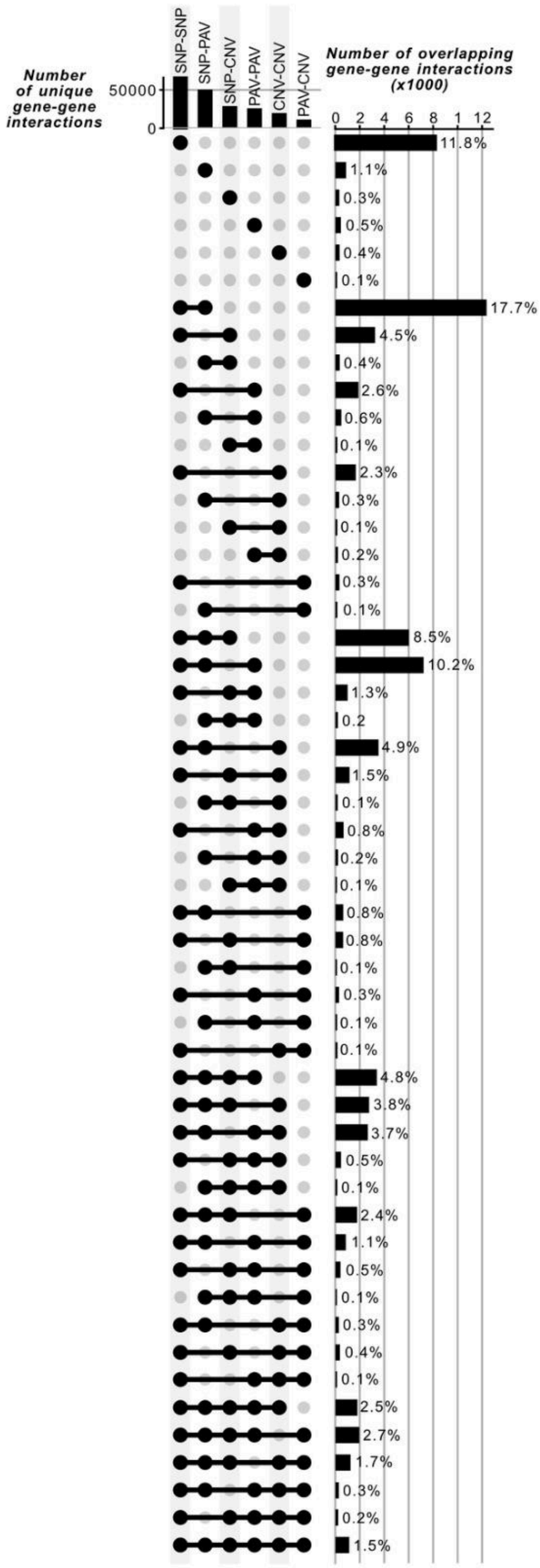

S12 Fig. Comparison of unique gene-gene interactions represented by different variant-variant SHAP interactions

The number of unique (top-left bar chart) and overlapping gene-gene interactions (right bar chart) identified by SHAP interactions for different variant pair types. For the right bar chart, a single black dot represents the number of gene-gene interactions identified by a variant-variant interaction type (i.e., SNP-SNP, PAV-PAV, CNV-CNV, SNP-PAV, SNP-CNV, and PAV-CNV). The black line connecting two or more dots represents the comparison that is being made (e.g., comparing the overlap between SNP-SNP and PAV-PAV gene-gene interactions). The percentages of overlapping gene-gene interactions out of the total number of gene-gene interactions (69,486) are shown.

Supplemental table legends

S1 Table. List of environmental conditions and estimates of genetic variation in the SNPs, PAVs, and CNVs.

The list of 35 environments from which fitness values were obtained, and measures of genetic variation in the SNP, PAV, and CNV feature sets, are shown. For SNPs, genetic variation is estimated as genotype frequencies and allele frequencies of the major and minor alleles, and for the reference and alternative alleles, based on the 118,382 SNPs across 750 isolates; as well as the number of SNPs found within the genic region of a gene. PAV variation is reported as the proportion of isolates (out of 750) in which an ORF is present. CNV variation is represented using descriptive statistics of CNV values across 750 isolates, including the mean, standard deviation, 25th, 50th (median), and 75th percentiles, and the variance.

S2 Table. Performances of single-environment models built using different algorithms and genetic variant features.

For each of the 35 environments, a single-environment fitness prediction model was trained using linear and non-linear algorithms. Model training consisted of hyperparameter tuning via 5-fold cross-validation, training the model with the best hyperparameters within a 5-fold cross-validation scheme, and evaluating the model on a held-out test set consisting of one-sixth of the yeast isolates (125 out of 750 isolates). See **Materials and Methods** for details on model training. Models were trained on the first five principal components of the SNP features (PCs), SNPs, PAVs, or CNVs. Feature selection was implemented using the RF models and the subsequent optimized feature sets were used to train XGBoost, rrBLUP, Bayesian LASSO, and BayesC models. Reported metrics include average validation and test set performances, measured by R^2 and Pearson's correlation coefficient.

S3 Table. Comparison of model performances by algorithm, environment, and genetic variant type.

Pearson correlation coefficients of model performances across the 35 single-environment RF models trained using different algorithms. Correlations were calculated between pairs of algorithms, pairs of environments across algorithms or genetic variant types, and pairs of genetic variant types used to train models. A Mann-Whitney U test was also performed for pairs of algorithms to determine which algorithm generally performed the best.

S4 Table. Contribution of fitness-related features and the number of features used to train models to RF model performances.

Linear regression analysis of the model performances of the 35 PC, SNP, PAV, or CNV optimized RF models against three fitness-related features: narrow-sense heritability (h^2), fitness variance, and median fitness in each environment, in addition to all pairwise and three-way interaction features (e.g., h^2-by-fitness variance, see **Materials and Methods**). All features were centered and scaled prior to regression. SHAP values for each feature were estimated using LinearExplainer (see **Materials and Methods**). The feature values and SHAP values are provided in separate sheets. In summary, the variance in fitness in the five best predicted environments ranges from 0.014 to 0.210, and in the remaining environments, the variance ranges from 0.006 to 0.087. Median fitness values ranged from 0.11 to 0.51 for the five best predicted environments and 0.11 to 0.96 for the remaining environments. Fitness variance-by-h^2 interaction term values ranged from 0.011 to 0.192 for the five best predicted environments and 0.003 to 0.055 for the remaining environments. For SNPs, the term with the smallest p-value was h^2 (P = 0.06, coefficient = 0.07). An additional linear regression analysis was conducted using only the number of features used to train the optimized RF models.

S5 Table. Comparison of feature ranks between Gini importance and SHAP values.

Spearman's rank correlation between Gini importance and average absolute SHAP values of features from single-environment RF models trained on SNPs, PAVs, or CNVs. Rho values, p-values, and the

number of overlapping features identified by both feature importance measures are reported for all 35 environments (see **Materials and Methods**).

S6 Table. Comparison of feature ranks between genetic variant types.

Spearman's rank correlation of Gini or SHAP-based feature importances between RF models trained on different genetic variant types (SNP vs PAV, SNP vs CNV, and PAV vs CNV). Comparisons were performed for both the RF models trained on either complete or optimized feature sets for all 35 environments. Rho values, *p*-values, and the number of overlapping genes identified by both feature importance measures are reported for all 35 environments (see **Materials and Methods**).

S7 Table. Comparison of feature ranks between environments.

Spearman's rank correlation (rho) of rankings based on Gini or average absolute SHAP feature importances between environments. Correlations were calculated separately for SNP, PAV, and CNV single-environment RF models trained on either the optimized or complete feature sets. The number of overlapping features between environments are provided in a separate sheet.

S8 Table. GO term and pathway enrichment analysis results.

Enrichment of GO terms and pathway annotations (see **Materials and Methods**) among genes from the optimized single-environment RF models built using SNP, PAV, or CNV features. Enrichment analyses were performed separately for each genetic variant type, and genes from the optimized models were compared to a background set consisting of non-overlapping genes represented by the full set of SNP, PAV, or CNV features. Intergenic SNPs were excluded. Annotations were considered significantly enriched if they had a $q < 0.05$ after multiple testing correction using the Benjamini-Hochberg method to control the false discovery rate.

S9 Table. SNP, PAV, and CNV feature importances.

Gini importances or average absolute SHAP values of SNP, PAV, and CNV features from the optimized RF models for all 35 environments. Feature-to-S288C gene mappings are provided, along

with GO term and pathway annotations and an indication of whether each gene is an SGD benchmark gene.

S10 Table. Lists of experimentally validated benchmark fitness genes.

Lists of benchmark genes experimentally proven to decrease fitness in the benomyl, caffeine, copper(II) sulfate, or sodium meta-arsenite environments. Benchmark genes were collected from SGD and manually curated from the literature.

S11 Table. Enrichment analysis of benchmark genes.

Enrichment analysis of benchmark genes from SGD for benomyl, caffeine, copper(II) sulfate, or sodium meta-arsenite stress and from the manually curated gene list. Results are reported for different rank percentile thresholds (ranks within the 1st, 5th, 10th, 15th, 20th, or 25th percentiles) for five environments: YPD Caffeine 40 mM, YPD Caffeine 50 mM, YPD Benomyl 500 µg/ml, YPD $CuSO_4$ 10 mM, and YPD Sodium meta-arsenite 2.5 mM.

S12 Table. Association between feature number and benchmark gene model performance, and comparisons of genetic variation between benchmark genes and important non-benchmark gene feature sets.

Analyses were conducted to evaluate whether differences in model performance between benchmark genes and important non-benchmark genes were attributable to feature set size and/or underlying genetic variation. A linear regression was performed using the number of features as the independent variable and the performance R^2 of the test set as the dependent variable. Reported in the sheet called "Feature number regression" are the slope, standard error of the slope, intercept, *p*-value of the hypothesis test (alternative hypothesis: the slope of the regression line is nonzero), and the Pearson's correlation coefficient. Genetic variation was compared between benchmark gene features and important non-benchmark gene features used to train the models shown in Fig 4A. Genetic variation was quantified in two ways: (1) the Euclidean distance of SNP, PAV, or CNV features across 625 isolates used for model training and (2) the percent presence of ORFs. Distributions of Euclidean

distances were compared using the Wilcoxon signed-rank test, with the alternative hypothesis that benchmark genes show less variation than important non-benchmark genes. Distributions of ORF percent presence were compared using Welch's t-tests, with the alternative hypothesis that benchmark genes are more frequently present than important non-benchmark genes. Results are reported in the sheets called "Euclidean distance comparison" and "Percent presence comparison".

S13 Table. Effect of genetic relatedness to the genetic background used for experimental validation of benchmark genes on feature importance.

SNP and PAV genotypes of the training isolates were compared to those of the laboratory strain S288C using Euclidean distance as a measure of genetic relatedness. K-means clustering was used to identify clusters of isolates. A Mann-Whitney U test was conducted to compare the median absolute SHAP values of benchmark genes between clusters of isolates. Results are reported for five environments: YPD Caffeine 40 mM, YPD Caffeine 50 mM, YPD Benomyl 500 μg/ml, YPD $CuSO_4$ 10 mM, and YPD Sodium meta-arsenite 2.5 mM. See **Materials and Methods** for details on measuring genetic relatedness, cluster analysis, deriving gene-level feature importance, and conducting the Mann-Whitney U test.

S14 Table. Correlation between fitness and SHAP values of isolates within a cluster for the top 20 predictive features, and relationships between CNV values, fitness, and CNV feature importances.

Isolates that were used to train optimized RF models were clustered based on the SHAP values of the top 20 features from the optimized SNP, PAV, or CNV RF models for five environments (YPD Caffeine 40 mM, YPD Caffeine 50 mM, YPD Benomyl 500 μg/ml, YPD $CuSO_4$ 10 mM, and YPD Sodium meta-arsenite 2.5 mM). Within each cluster, the Pearson correlation coefficient between median absolute SHAP values, which was calculated across the top 20 features, and median fitness of isolates was quantified and a linear regression was fitted. Results are reported in the sheet called "top20_shap_vs_fitness". To assess the relationship between isolate fitness and CNV values of features from the optimized RF models, Pearson correlations were calculated. Pearson correlation

coefficients and p-values are reported in the sheet “cnv_vs_fitness_corr”. To evaluate the extent to which the CNV-fitness correlations were associated with feature importance, the CNV-fitness correlations were binned according to the median absolute SHAP values. A linear regression line was fitted across the median CNV-fitness correlations of the median absolute SHAP value bins.

S15 Table. ORF to S288C gene mappings based on BLAST.

Table containing the systematic identifiers of S288C genes that were mapped to ORF features.
For each ORF, the minimum E-value across BLASTx and tBLASTx alignments and the average percent identity are reported.

S16 Table. Performance of models used to obtain SHAP interaction values and its association with feature number.

Performance of RF models built using reduced SNP, PAV, CNV, or integrated feature sets for predicting fitness in the YPD Benomyl 500 μg/mL environment (see **Estimating SHAP interaction values**). A linear regression line was fitted to the model performances on the number of features used to train models. Linear regression results are provided in a separate sheet.

S17 Table. Gene-gene interactions identified by the SHAP-based feature interactions.

SHAP interaction values from YPD Benomyl 500 μg/mL RF models trained using the reduced SNP, CNV, or integrated feature sets. SHAP interactions from the PAV model were excluded because of the poor performance on the test set.

S18 Table. Experimentally validated gene-gene interactions identified by different variant-variant interaction types.

The list of variant-variant feature interaction types are listed for each gene pair with experimental validation information.

S19 Table. Enrichment of experimentally validated genetic interactions.

Enrichment of experimentally validated genetic interactions in the SHAP interactions from the six RF models trained using the SNP, CNV, SNP + PAV, SNP + CNV, PAV + CNV, or SNP + PAV + CNV feature sets. Enrichment was assessed within the 1st, 5th, 10th, 15th, 20th, and 25th rank percentiles based on SHAP interaction values for each model.

Supplemental file legends

S1 File. Fitness measurements.

Fitness in 35 environments for 750 diploid *Saccharomyces cerevisiae* isolates.

S2 File. SNP genotypes.

Filtered SNP matrix containing 118,382 bi-allelic SNPs, which are encoded as -1, 0, or 1.

S3 File. Kinship matrix.

Kinship matrix from the SNP data.

S4 File. PC matrix.

First five principal components of the SNP data.The variance explained by each principal component is also provided.

S5 File. PAV matrix.

Filtered presence/absence variant data containing 7,708 ORFs.

S6 File. CNV matrix.

Filtered copy number variant data containing 7,708 ORFs.

S7 File. Feature importances from the RF models built using complete feature sets.

Gini importance and average absolute SHAP values of SNP, PAV, and CNV features from the single-environment RF models built using complete feature sets.

S8 File. SNP-to-gene map.

Table containing the systematic identifiers of S288C genes that were mapped to SNP features. Four additional columns indicate whether the S288C gene is a benchmark fitness gene for benomyl, caffeine, $CuSO_4$, and/or sodium meta-arsenite.

S9 File. ORF-to-gene map.

Table containing the systematic identifiers of S288C genes that were mapped to ORFs. Four additional columns indicate whether the S288C gene is a benchmark fitness gene for benomyl, caffeine, $CuSO_4$, and/or sodium meta-arsenite.

S10 File. Experimentally validated genetic interactions.

The combined set of 441,520 unique experimentally verified genetic interactions from the BioGRID database and the benomyl and control condition networks from Costanzo et al., 2016.

S11 File. Performances of benchmark gene models.

The number of features and validation/testing performance R^2 values of RF models trained using benchmark gene sets, important non-benchmark gene sets, and combined benchmark + important non-benchmark gene sets for the YPD Caffeine 40 mM, YPD Caffeine 50 mM, YPD Benomyl 500 µg/mL, YPD $CuSO_4$ 10 mM, and YPD Sodium meta-arsenite 2.5 mM environments (see **Assessing the contribution of benchmark genes to fitness predictions** for details).

S12 File. SNP genotypes with S288C genotypes included.

118,382 bi-allelic SNP genotypes encoded as 0 (homozygous for the reference allele), 1 (heterozygous), and 2 (homozygous for the alternative allele). S288C SNP genotypes were encoded as a vector of 0s.

S13 File. Euclidean distance of the SNP genotypes.

Genetic distances between the 625 training isolates and S288C, which were calculated using Euclidean distance of the SNP genotype matrix.

S14 File. Euclidean distance of the PAV genotypes.

Genetic distances between the 625 training isolates and S288C, which were calculated using Euclidean distance of the PAV genotype matrix.

S15 File. SHAP cluster distance thresholds.

Distance thresholds used to define isolate clusters at different granularities, based on the SHAP values of the top 20 SNP, PAV, and CNV features from the optimized YPD Caffeine 40 mM, YPD Caffeine 50 mM, YPD Benomyl 500 µg/mL, YPD $CuSO_4$ 10 mM, and YPD Sodium meta-arsenite 2.5 mM RF models.

S16 File. SHAP values of isolates clustered based on the top 20 important features.

SHAP values of the top 20 SNP, PAV, and CNV features from the optimized RF models for YPD Caffeine 40 mM, YPD Caffeine 50 mM, YPD Benomyl 500 µg/mL, YPD $CuSO_4$ 10 mM, and YPD Sodium meta-arsenite 2.5 mM. The row and column ordering of each SHAP value matrix corresponds to the heatmaps shown in Figs 5, S6, S7, S8, S9, and S10.
